# Unconventional Thermal and Magnetic-Field-Driven Changes of a Bipartite Entanglement of a Mixed Spin-(1/2,*S*) Heisenberg Dimer with an Uniaxial Single-Ion Anisotropy

**DOI:** 10.3390/nano11113096

**Published:** 2021-11-16

**Authors:** Hana Vargová, Jozef Strečka

**Affiliations:** 1 Institute of Experimental Physics, Slovak Academy of Sciences, Watsonova 47, 040 01 Košice, Slovakia; hcencar@saske.sk; 2 Department of Theoretical Physics and Astrophysics, Faculty of Science, P. J. Šafárik University, Park Angelinum 9, 040 01 Košice, Slovakia

**Keywords:** quantum and thermal entanglement, spin-(1/2,S) Heisenberg dimer, exact calculations, uniaxial single-ion anisotropy

## Abstract

The concept of negativity is adapted in order to explore the quantum and thermal entanglement of the mixed spin-(1/2,*S*) Heisenberg dimers in presence of an external magnetic field. The mutual interplay between the spin size *S*, XXZ exchange and uniaxial single-ion anisotropy is thoroughly examined with a goal to tune the degree and thermal stability of the pairwise entanglement. It turns out that the antiferromagnetic spin-(1/2,*S*) Heisenberg dimers exhibit higher degree of entanglement and higher threshold temperature in comparison with their ferromagnetic counterparts when assuming the same set of model parameters. The increasing spin magnitude *S* accompanied with an easy-plane uniaxial single-ion anisotropy can enhance not only the thermal stability but simultaneously the degree of entanglement. It is additionally shown that the further enhancement of a bipartite entanglement can be achieved in the mixed spin-(1/2,*S*) Heisenberg dimers, involving half-odd-integer spins *S*. Under this condition the thermal negativity saturates at low-enough temperatures in its maximal value regardless of the magnitude of half-odd-integer spin *S*. The magnetic field induces consecutive discontinuous phase transitions in the mixed spin-(1/2,*S*) Heisenberg dimers with S>1, which are manifested in a surprising oscillating magnetic-field dependence of the negativity observed at low enough temperature.

## 1. Introduction

Extraordinary correlations between subsystems of a quantum-mechanical ensemble, known as entanglement, belongs to the most fascinating phenomena attracting a lot of attention during the last few decades. A huge concerment in this field of study closely relates to perspective applications of this phenomenon in a quantum computing [1,2,3,4], quantum information [5,6], and quantum memory circuits decoding [7,8,9]. However, a long period before, it was believed that the entanglement could exist exclusively on the atomic scale and completely vanishes at macroscopic scales as a consequence of the decoherence arising from the interactions between a large number of matter constituents and their environment. It was presumed, in addition, that arbitrarily small thermal fluctuations rapidly smear out the quantum correlations and thus, the entanglement cannot exist at non-zero temperatures. Some theoretical predictions [10,11,12,13,14,15] have presented relevant arguments that the entanglement could surprisingly exist even at finite temperatures, but rapidly falls down as temperature increases.

From a theoretical perspective low-dimensional Heisenberg spin models seem to be a reasonable theoretical ground, which allows an exact study of the quantum and thermal entanglement depending on external stimuli such as the magnetic field and/or temperature [16,17,18,19,20,21,22,23,24,25,26,27,28]. Besides the most intensively analysed spin-1/2 case, a few theoretical works were focused on the entanglement of the mixed-spin Heisenberg systems [26,27,28,29,30,31,32,33,34,35,36,37,38,39,40,41,42,43,44,45,46,47,48]. The particular interest in this field of study has been motivated by the pioneering work by Wang et al. [26], which demonstrated that a higher difference between dissimilar spin constituents can slightly enhance the thermal entanglement at higher temperatures due to the respective shift of its threshold temperature. Unfortunately, the enlargement of a threshold temperature is simultaneously accompanied by the reduction of the strength of mutual quantum correlations. In order to minimize the reduction of the degree of entanglement, many of subsequent studies were concentrated on an extended mixed-spin Heisenberg chain involving the Dzyaloshinskii–Moriya interaction (DMI) [34,35,36,37], the effect of nonuniform magnetic field [37,38,39,40,41,42,43], long-range interaction [44] and uniaxial single-ion anisotropy [45,46], respectively. It was verified for the mixed spin-(1/2,1), spin-(1/2,3/2) and spin-(1/2,5/2) Heisenberg chains that the inhomogeneity of the external magnetic field can be suitable tuning parameter for enhancing the thermal entanglement in a high-temperature region. On the other hand, the DMI can enhance the low-temperature entanglement of the antiferromagnetic mixed spin-(1/2,3/2) Heisenberg dimer [36], nevertheless the enhancement of entanglement in the mixed spin-(1/2,1) Heisenberg dimer is possible only for the ferromagnetic exchange coupling [34]. Based on the results obtained for the mixed spin-(1/2,1) Heisenberg dimer [45,46], the uniaxial single-ion anisotropy seems to be another relevant driving force for an enhancement of entanglement in presence of thermal fluctuations. However, the comprehensive analysis of other mixed-spin Heisenberg dimers with higher spins S>1 is still absent. This fact motivated us to study the mixed spin-(1/2,*S*) Heisenberg dimers involving the uniaxial single-ion anisotropy and magnetic field simultaneously with the special goal to verify whether the interplay between the uniaxial single-ion anisotropy, magnetic field and the spin magnitude *S* can enhance thermal entanglement at high enough temperatures. It should be emphasized that the uniaxial single-ion anisotropy may be relevant for several heterodinuclear complexes as for instance in magnetic compounds [49] such as the [MnCu(pbaOH)(H${}_{2}$O)${}_{3}$]·nH${}_{2}$O (pbaOH = 2-hydroxy-1,3-propylenebis (oxamato)) [50], [Ni(dpt)(H${}_{2}$O)${}_{3}$Cu(pba)]·H${}_{2}$O (pba = 1,3-propylenebis(oxamato) and dpt = bis-(3-aminopropyl)amine) [51] or [NiCu(pba)(D${}_{2}$O)${}_{3}$]·2D${}_{2}$O [52].

The paper is organized as follows. The investigated mixed spin-(1/2,*S*) Heisenberg dimer will be defined in Section 2 together with a few details of the calculation procedure used in our rigorous study. The most interesting results concerned with the quantum and thermal entanglement under the influence of increasing spin magnitude *S* will be discussed in Section 3. Besides the effect of an applied external magnetic field on the thermal entanglement and the threshold temperature of the mixed spin-(1/2,*S*) Heisenberg dimers will be also discussed for an arbitrary spin-*S*. Finally, some concluding remarks are given in Section 4 and a few details of analytical derivations are presented in Appendix A, Appendix B and Appendix C.

## 2. Model and Method

Let us consider the mixed spin-(1/2,*S*) Heisenberg dimers with an uniaxial single-ion anisotropy under the influence of the external magnetic field defined through the following Hamiltonian:

(1) $$\begin{array}{ccc} \hat{H}\;\;\; & = & \;\;\;J\left[\Delta \left({\hat{\mu }}^{x}{\hat{S}}^{x}\;+\;{\hat{\mu }}^{y}{\hat{S}}^{y}\right)\;+\;{\hat{\mu }}^{z}{\hat{S}}^{z}\right]\;+\;D{\left({\hat{S}}^{z}\right)}^{2}\;-\;Bg\mu_{B}\left({\hat{\mu }}^{z}\;+\;{\hat{S}}^{z}\right). \end{array}$$

In above, the symbols ${\hat{\mu }}^{\alpha }$ and ${\hat{S}}^{\alpha }$ (α=x,y,z) correspond to spatial components of spin-1/2 and spin-*S* (S≥1) operators, *J* is the XXZ exchange interaction with an exchange anisotropy Δ, *D* is the uniaxial single-ion anisotropy acting on the spin-*S* magnetic ion only. Finally, the model under the investigation accounts for the effect of external magnetic field *B* applied along the *z*-direction, *g* denotes the gyromagnetic Landé *g*-factor and $\mu_{B}$ is the Bohr magneton.

In order to study the quantum and thermal entanglement of the mixed spin-(1/2,*S*) Heisenberg dimers we will employ the concept of Peres–Horodecki [53,54], according to which negative eigenvalue of a partially transposed density matrix is a necessary condition for the onset of entanglement. To quantify the strength of quantum and thermal entanglement one may therefore utilize the quantity known as negativity [55]. The negativity of the mixed state given by the density matrix ρ is by definition the sum of all negative eigenvalues $\lambda_{i}$ of partially transposed density matrix ${\hat{\rho }}^{T_{1/2}}$

(2) $$\begin{array}{c} N\left(\rho \right)\;=\;\sum_{\lambda_{i}<0}\left|\lambda_{i}\right|. \end{array}$$

It is worthwhile to remark that the negativity of the maximally entangled state is equal to one-half (N=1/2) for the mixed spin-(1/2,*S*) Heisenberg dimers, whereas the negativity completely vanishes (N=0) in the separable states without the bipartite entanglement.

Before calculating the negativity it is necessary to derive the eigenvalues and eigenvectors of the Hamiltonian (1), which can be easily calculated in the standard orthonormal basis $|\mu^{z},S^{z}\rangle$ constructed from all available eigenvectors of *z*-components of two constituent spins with eigenvalues $\mu^{z}\;=\;\pm 1/2$ and $S^{z}\;=\;-S,-S\;+\;1,\ldots ,S\;-\;1,S$. For this purpose, let us introduce first the notation for raising and lowering ladder operators ${\hat{S}}^{\pm }\;=\;{\hat{S}}^{x}\;\pm \;i{\hat{S}}^{y}$ and ${\hat{\mu }}^{\pm }\;=\;{\hat{\mu }}^{x}\;\pm \;i{\hat{\mu }}^{y}$, which allows us to rewrite the Hamiltonian (1) into the more convenient form

(3) $$\begin{array}{cc} \hat{H} & \;=\;J\left[\frac{\Delta }{2}\left({\hat{S}}^{+}{\hat{\mu }}^{-}\;+\;{\hat{S}}^{-}{\hat{\mu }}^{+}\right)\;+\;{\hat{S}}^{z}{\hat{\mu }}^{z}\right]\;+\;D{\left({\hat{S}}^{z}\right)}^{2}\;-\;Bg\mu_{B}\left({\hat{\mu }}^{z}\;+\;{\hat{S}}^{z}\right). \end{array}$$

As a result, one immediately realizes that the *z*-component of the spin operators ${\hat{S}}^{z}$ (${\hat{\mu }}^{z}$) trivially act on the original basis states ${\hat{S}}^{z}|S^{z}\rangle \;=\;S^{z}|S^{z}\rangle$ and ${\hat{\mu }}^{z}|\mu^{z}\rangle \;=\;\mu^{z}|\mu^{z}\rangle$, whereas the raising and lowering ladder operators ${\hat{S}}^{+}$ (${\hat{\mu }}^{+}$) and ${\hat{S}}^{-}$ (${\hat{\mu }}^{-}$) shift the relevant quantum spin number by unity

(4) $$\begin{array}{ccc} \; & {\hat{S}}^{\mp }|S^{z}\rangle \;=\;\sqrt{S\left(S\;+\;1\right)\;-\;S^{z}\left(S^{z}\;\mp \;1\right)}|S^{z}\;\mp \;1\rangle ,\;\; & {\hat{\mu }}^{\mp }|\mu^{z}\rangle \;=\;\sqrt{\frac{3}{4}\;-\;\mu^{z}\left(\mu^{z}\;\mp \;1\right)}|\mu^{z}\;\mp \;1\rangle . \end{array}$$

Subsequently, the application of the Hamiltonian (3) on the basis state $|\pm 1/2,S^{z}\rangle$ leads to the identity

(5) $$\begin{array}{cc} \hat{H}|\pm \frac{1}{2},S^{z}\rangle & \;=\;\frac{J\Delta }{2}\sqrt{S\left(S\;+\;1\right)\;-\;S^{z}\left(S^{z}\;\pm \;1\right)}\;|\mp \frac{1}{2},S^{z}\;\pm \;1\rangle \\ \; & \;+\;\frac{1}{2}\left[S^{z}\left(\pm J\;+\;2DS^{z}\right)\;-\;h\left(2S^{z}\;\pm \;1\right)\right]|\pm \frac{1}{2},S^{z}\rangle , \end{array}$$

where $S^{z}\;=\;-S,-S\;+\;1,\ldots ,S\;-\;1,S$ and $h\;=\;Bg\mu_{B}$. The non-zero matrix elements define the block diagonal structure of the Hamiltonian, which consists of two one-by-one blocks and 2*S* two-by-two blocks characterized by a specific value of the *z*-component of the total spin $S_{t}^{z}\;=\;S^{z}\;+\;\mu^{z}$ running from −S−1/2 to S+1/2. Consequently, one can easily derive the respective eigenvalues and eigenvectors. The extremal values of $S_{t}^{z}\;=\;\pm \left(S\;+\;1/2\right)$ define two one-by-one blocks, whose element is identical with its eigenvalue and the respective standard basis state designates the corresponding eigenvector

(6) $$\begin{array}{cc} \langle \pm \frac{1}{2},\pm S|\hat{H}|\pm \frac{1}{2},\pm S\rangle & \;=\;\frac{1}{2}\left[S(J\;+\;2DS)\;\mp \;h(2S\;+\;1)\right],\\ \varepsilon_{S,\pm (S+\frac{1}{2})} & \;=\;\frac{1}{2}\left[S(J\;+\;2DS)\;\mp \;h(2S\;+\;1)\right],\\ |\pm \left(S\;+\;\frac{1}{2}\right)\rangle & \;=\;|\pm \frac{1}{2},\pm S\rangle . \end{array}$$

The remaining sectors with the total spin momentum $S_{t}^{z}\;=\;-S\;+\;1/2,-S\;+\;3/2,\ldots ,S\;-\;1/2$ ($S^{z}\;=\;-S\;+\;1,\ldots ,S\;-\;1$) form two-by-two blocks

(7) $$\begin{array}{cc} \; & \left(\begin{array}{cc} \langle \frac{1}{2},S^{z}|\hat{H}|\frac{1}{2},S^{z}\rangle & \langle \frac{1}{2},S^{z}|\hat{H}|\;-\;\frac{1}{2},S^{z}\;+\;1\rangle \\ \langle \;-\;\frac{1}{2},S^{z}\;+\;1|\hat{H}|\frac{1}{2},S^{z}\rangle & \langle \;-\;\frac{1}{2},S^{z}\;+\;1|\hat{H}|\;-\;\frac{1}{2},S^{z}\;+\;1\rangle \end{array}\right) \end{array}$$

with the matrix elements explicitly defined as

(8) $$\begin{array}{cc} \; & \langle \frac{1}{2},S^{z}|\hat{H}|\frac{1}{2},S^{z}\rangle \;=\;\frac{1}{2}\left[S^{z}\left(J\;+\;2DS^{z}\right)\;-\;h\left(2S^{z}\;+\;1\right)\right],\\ \; & \langle -\frac{1}{2},S^{z}\;+\;1|\hat{H}|-\frac{1}{2},S^{z}\;+\;1\rangle \;=\;\frac{1}{2}\left[-\left(S^{z}\;+\;1\right)\left(J\;-\;2DS^{z}\;-\;2D\right)\;-\;h\left(2S^{z}\;+\;1\right)\right],\\ \; & \langle \frac{1}{2},S^{z}|\hat{H}|-\frac{1}{2},S^{z}\;+\;1\rangle \;=\;\langle -\frac{1}{2},S^{z}\;+\;1|\hat{H}|\frac{1}{2},S^{z}\rangle \;=\;\frac{J\Delta }{2}\sqrt{S\left(S\;+\;1\right)\;-\;S^{z}\left(S^{z}\;+\;1\right)}. \end{array}$$

The respective couples of eigenvalues and eigenvectors within those two-by-two orthogonal subspaces read

(9) $$\begin{array}{cc} \varepsilon_{S,S_{t}^{z}}^{\mp } & =-\frac{P_{S_{t}^{z}}}{4}\;\mp \;\frac{1}{4}\sqrt{R_{S_{t}^{z}}^{2}\;+\;Q_{S,S_{t}^{z}}},\;|{\left(S_{t}^{z}\right)}_{\mp }\rangle =c_{S,S_{t}^{z}}^{\mp }|\frac{1}{2},S^{z}\rangle \;\mp \;c_{S,S_{t}^{z}}^{\pm }|\;-\frac{1}{2},S^{z}\;+\;1\rangle . \end{array}$$

For brevity, we have introduced in Equation (9) the new functions $P_{S_{t}^{z}}$, $R_{S_{t}^{z}}$, $Q_{S,S_{t}^{z}}$, $c_{S,S_{t}^{z}}^{\mp }$ denoting the following expressions

(10) $$\begin{array}{cc} \; & P_{S_{t}^{z}}=\left(J\;-\;2D\right)\;-\;D\left(2S_{t}^{z}\;-\;1\right)\left(2S_{t}^{z}\;+\;1\right)\;+\;4hS_{t}^{z}, \end{array}$$

(11) $$\begin{array}{cc} \; & R_{S_{t}^{z}}=2\left(J\;-\;2D\right)S_{t}^{z}, \end{array}$$

(12) $$\begin{array}{cc} \; & Q_{S,S_{t}^{z}}={\left(J\Delta \right)}^{2}\left[4S\left(S\;+\;1\right)\;-\;\left(2S_{t}^{z}\;-\;1\right)\left(2S_{t}^{z}\;+\;1\right)\right], \end{array}$$

(13) $$\begin{array}{cc} \; & c_{S,S_{t}^{z}}^{\mp }\;=\;\frac{1}{\sqrt{2}}\sqrt{1\;\mp \;\frac{R_{S_{t}^{z}}}{\sqrt{R_{S_{t}^{z}}^{2}\;+\;Q_{S,S_{t}^{z}}}}\;}. \end{array}$$

Based on the knowledge of a complete energy spectrum of eigenvalues $\varepsilon_{S,\pm (S+1/2)},\varepsilon_{S,S_{t}^{z}}^{\mp }$ and corresponding eigenvectors $\left|\pm \left(S\;+\;1/2\right)\rangle ,\right|{\left(S_{t}^{z}\right)}_{\mp }\rangle$ ($S_{t}^{z}\;=\;-S\;+\;1/2,\ldots ,S\;-\;1/2$), one is able to construct the relevant density operator $\hat{\rho }$ according to the formula

(14) $$\begin{array}{cc} \hat{\rho } & =\frac{1}{Z}\left\{e^{-\beta \varepsilon_{S,S+\frac{1}{2}}}|S\;+\;\frac{1}{2}\rangle \langle S\;+\;\frac{1}{2}|\right.\;+\;e^{-\beta \varepsilon_{S,-(S+\frac{1}{2})}}|-\left(S\;+\;\frac{1}{2}\right)\rangle \langle -\left(S\;+\;\frac{1}{2}\right)|\\ \; & \;+\;\sum_{S_{t}^{z}=-S+1/2}^{S-1/2}\left[e^{-\beta \varepsilon_{S,S_{t}^{z}}^{-}}|{\left(S_{t}^{z}\right)}_{-}\rangle \langle {\left(S_{t}^{z}\right)}_{-}|\right.\;+\;\left.\left.e^{-\beta \varepsilon_{S,S_{t}^{z}}^{+}}|{\left(S_{t}^{z}\right)}_{+}\rangle \langle {\left(S_{t}^{z}\right)}_{+}|\right]\right\}, \end{array}$$

where $\beta \;=\;1/(k_{B}T)$, *T* is an absolute temperature, $k_{B}$ is a Boltzmann’s constant and Z is the partition function

(15) $$\begin{array}{cc} Z & \;=\;2\left\{e^{-\frac{\beta S}{2}\left(J+2DS\right)}\cosh\left[\frac{\beta h}{2}\left(2S\;+\;1\right)\right]\right.\;+\;\left.\sum_{S_{t}^{z}=-S+1/2}^{S-1/2}\;\;e^{\frac{\beta }{4}P_{S_{t}^{z}}}\cosh\left(\frac{\beta }{4}\sqrt{R_{S_{t}^{z}}^{2}\;+\;Q_{S,S_{t}^{z}}}\right)\right\}. \end{array}$$

The density matrix $\hat{\rho }$ representing a matrix representation of the density operator (14) again has in the standard basis the same block diagonal form (classified according to the $S_{t}^{z}$ value) involving two one-by-one blocks with the extremal values of the total spin $S_{t}^{z}\;=\;\pm \left(S\;+\;1/2\right)$ and 2*S* two-by-two blocks with $S_{t}^{z}\;=\;-S\;+\;1/2,\ldots ,S\;-\;1/2$. All non-zero elements of the density matrix can be commonly expressed through the following general formulas

(16) $$\begin{array}{cc} \; & \langle \frac{1}{2},S^{z}|\hat{\rho }|\frac{1}{2},S^{z}\rangle \;=\;=\;\left\{\begin{array}{cc} \frac{1}{Z}e^{-\beta \varepsilon_{S,S+\frac{1}{2}}}, & \;if\;\;S^{z}\;=\;S;S_{t}^{z}\;=\;S\;+\;\frac{1}{2};\\ \frac{1}{Z}\left[{\left(c_{S,S_{t}^{z}}^{-}\right)}^{2}e^{-\beta \varepsilon_{S,S_{t}^{z}}^{-}}\;+\;{\left(c_{S,S_{t}^{z}}^{+}\right)}^{2}e^{-\beta \varepsilon_{S,S_{t}^{z}}^{+}}\right], & \\ \; & \;if\;\;S^{z}\;=\;-S,-S\;+\;1,\ldots ,S\;-\;1,\\ \; & \;\;\;\;\;S_{t}^{z}\;=\;-S\;+\;\frac{1}{2},\ldots ,S\;-\;\frac{1}{2}, \end{array}\right. \end{array}$$

(17) $$\begin{array}{cc} \; & \langle -\frac{1}{2},S^{z}|\hat{\rho }|-\frac{1}{2},S^{z}\rangle \;=\;=\;\left\{\begin{array}{cc} \frac{1}{Z}e^{-\beta \varepsilon_{S,-(S+\frac{1}{2})}}, & \;if\;\;S^{z}\;=\;-S;S_{t}^{z}\;=\;-S\;-\;\frac{1}{2};\\ \frac{1}{Z}\left[{\left(c_{S,S_{t}^{z}}^{+}\right)}^{2}e^{-\beta \varepsilon_{S,S_{t}^{z}}^{-}}\;+\;{\left(c_{S,S_{t}^{z}}^{-}\right)}^{2}e^{-\beta \varepsilon_{S,S_{t}^{z}}^{+}}\right], & \\ \; & \;if\;\;S^{z}\;=\;-S\;+\;1,-S\;+\;2,\ldots ,S;S_{t}^{z}\;=\;S^{z}\;-\;\frac{1}{2},\\ \; & \;\;\;\;\;S_{t}^{z}\;=\;-S\;+\;\frac{1}{2},\ldots ,S\;-\;\frac{1}{2}, \end{array}\right.\\ \; & \langle \frac{1}{2},S^{z}|\hat{\rho }|-\frac{1}{2},S^{z}\;+\;1\rangle \;=\;\langle -\frac{1}{2},S^{z}\;+\;1|\hat{\rho }|\frac{1}{2},S^{z}\rangle \;=\;\frac{c_{S,S_{t}^{z}}^{-}c_{S,S_{t}^{z}}^{+}}{Z}\left[e^{-\beta \varepsilon_{S,S_{t}^{z}}^{+}}\;-\;e^{-\beta \varepsilon_{S,S_{t}^{z}}^{-}}\right],\\ \; & \;if\;\;S^{z}\;=\;-S,-S\;+\;1,\ldots ,S\;-\;1, \end{array}$$

(18) $$\begin{array}{cc} \; & \;\;\;\;\;S_{t}^{z}\;=\;-S\;+\;\frac{1}{2},\ldots ,S\;-\;\frac{1}{2}. \end{array}$$

For a completeness, the readers can find the explicit form of the density-matrix elements for a few selected mixed spin-(1/2,*S*) Heisenberg dimers (S=1,3/2,2,5/2) in the Appendix A.

In order to calculate the density matrix ${\hat{\rho }}^{T_{1/2}}$ partially transposed with respect to the spin-1/2 subsystem, it is sufficient to replace the bra and ket state vectors referred to the spin-1/2 subsystem. It is clear, that the diagonal elements derived from Equations (16) and (17) remain unchanged, whereas off-diagonal ones are moved to other positions. The partial transposition does not conserve the total spin momentum $S_{t}^{z}$, but it conserves the staggered spin momentum $S_{tm}^{z}\;=\;S^{z}\;-\;\mu^{z}$ running from −S−1/2 to S+1/2. Subsequently, the non-zero elements of the partially transposed density matrix ${\hat{\rho }}^{T_{1/2}}$ expressed in term of $S_{tm}^{z}$ have the form

(19) $$\begin{array}{cc} \; & \langle \frac{1}{2},S^{z}|{\hat{\rho }}^{T_{1/2}}|\frac{1}{2},S^{z}\rangle \;=\;\;\left\{\begin{array}{cc} \frac{1}{Z}e^{-\beta \varepsilon_{S,S+\frac{1}{2}}}, & \;if\;\;S^{z}\;=\;S;S_{tm}^{z}\;=\;S\;-\;\frac{1}{2}\\ \frac{1}{Z}\left[{\left(c_{S,S_{tm}^{z}+1}^{-}\right)}^{2}e^{-\beta \varepsilon_{S,S_{tm}^{z}+1}^{-}}\;+\;{\left(c_{S,S_{tm}^{z}+1}^{+}\right)}^{2}e^{-\beta \varepsilon_{S,S_{tm}^{z}+1}^{+}}\right], & \\ \; & \;if\;\;S^{z}\;=\;-S,-S\;+\;1,\ldots ,S\;-\;1,\\ \; & \;\;\;\;\;S_{tm}^{z}\;=\;-S\;-\;\frac{1}{2},\ldots ,S\;-\;\frac{3}{2}, \end{array}\right. \end{array}$$

(20) $$\begin{array}{cc} \; & \langle -\frac{1}{2},S^{z}|{\hat{\rho }}^{T_{1/2}}|-\frac{1}{2},S^{z}\rangle \;=\;\;\left\{\begin{array}{cc} \frac{1}{Z}e^{-\beta \varepsilon_{S,-(S+\frac{1}{2})}}, & \;if\;\;S^{z}\;=\;-S;S_{tm}^{z}\;=\;-S\;+\;\frac{1}{2}\\ \frac{1}{Z}\left[{\left(c_{S,S_{tm}^{z}-1}^{+}\right)}^{2}e^{-\beta \varepsilon_{S,S_{tm}^{z}-1}^{-}}\;+\;{\left(c_{S,S_{tm}^{z}-1}^{-}\right)}^{2}e^{-\beta \varepsilon_{S,S_{tm}^{z}-1}^{+}}\right], & \\ \; & \;if\;\;S^{z}\;=\;-S\;+\;1,-S\;+\;2,\ldots ,S,\\ \; & \;\;\;\;\;S_{tm}^{z}\;=\;-S\;+\;\frac{3}{2},\ldots ,S\;+\;\frac{1}{2}, \end{array}\right.\\ \; & \langle -\frac{1}{2},S^{z}|{\hat{\rho }}^{T_{1/2}}|\frac{1}{2},S^{z}\;+\;1\rangle \;=\;\langle \frac{1}{2},S^{z}\;+\;1|{\hat{\rho }}^{T_{1/2}}|-\frac{1}{2},S^{z}\rangle \;=\;\frac{c_{S,S_{tm}^{z}}^{-}c_{S,S_{tm}^{z}}^{+}}{Z}\left[e^{-\beta \varepsilon_{S,S_{tm}^{z}}^{+}}\;-\;e^{-\beta \varepsilon_{S,S_{tm}^{z}}^{-}}\right],\\ \; & \;if\;\;S^{z}\;=\;-S,-S\;+\;1,\ldots ,S\;-\;1, \end{array}$$

(21) $$\begin{array}{cc} \; & \;\;\;\;\;S_{tm}^{z}\;=\;-S\;+\;\frac{1}{2},\ldots ,S\;-\;\frac{1}{2}. \end{array}$$

Note that the density matrix ${\hat{\rho }}^{T_{1/2}}$ is a block diagonal with a maximal block’s size of 2 × 2 achieving a specific $S_{tm}^{z}$ value. Two one-by-one blocks with extremal $S_{tm}^{z}\;=\;\pm \left(S\;+\;1/2\right)$ involve a single element

(22) $$\begin{array}{cc} \; & \langle \pm \frac{1}{2},\mp S|{\hat{\rho }}^{T_{1/2}}|\pm \frac{1}{2},\mp S\rangle \;=\;\frac{1}{Z}\left[{\left(c_{S,\mp (S-\frac{1}{2})}^{\mp }\right)}^{2}e^{-\beta \varepsilon_{S,\mp (S-\frac{1}{2})}^{-}}\right.\left.\;+\;{\left(c_{S,\mp (S-\frac{1}{2})}^{\pm }\right)}^{2}e^{-\beta \varepsilon_{S,\mp (S-\frac{1}{2})}^{+}}\right],\; \end{array}$$

which directly determines two positive eigenvalues $\lambda_{\mp (S\;+\;1/2)}$ of a partially transposed density matrix

(23) $$\begin{array}{cc} \lambda_{\mp (S+\frac{1}{2})}\;=\;\frac{1}{Z} & \left[{\left(c_{S,\mp (S-\frac{1}{2})}^{\mp }\right)}^{2}e^{-\beta \varepsilon_{S,\mp (S-\frac{1}{2})}^{-}}\right.\;+\;\left.{\left(c_{S,\mp (S-\frac{1}{2})}^{\pm }\right)}^{2}e^{-\beta \varepsilon_{S,\mp (S-\frac{1}{2})}^{+}}\right]. \end{array}$$

Other 2*S* two-by-two blocks determined by basis state vectors with $S_{tm}^{z}\;=\;-S\;+\;1/2,\ldots ,S\;-\;1/2$

(24) $$\begin{array}{c} M\left(S_{tm}^{z}\right)\;\;=\;\;\left(\;\begin{array}{cc} \langle \frac{1}{2},S^{z}|{\hat{\rho }}^{T_{1/2}}|\frac{1}{2},S^{z}\rangle & \langle \frac{1}{2},S^{z}|{\hat{\rho }}^{T_{1/2}}|-\frac{1}{2},S^{z}\;-\;1\rangle \\ \langle -\frac{1}{2},S^{z}\;-\;1|{\hat{\rho }}^{T_{1/2}}|\frac{1}{2},S^{z}\rangle & \langle -\frac{1}{2},S^{z}\;-\;1|{\hat{\rho }}^{T_{1/2}}|-\frac{1}{2},S^{z}\;-\;1\rangle \end{array}\;\right)\;\;=\;\;\left(\;\begin{array}{cc} m_{11} & m_{12}\\ m_{21} & m_{22} \end{array}\;\right) \end{array}$$

immediately result to the remaining couple of eigenvalues

(25) $$\begin{array}{c} \;\lambda_{S_{tm}^{z}}^{\mp }\;=\;\frac{1}{2}\left[\left(m_{11}\;+\;m_{22}\right)\;\mp \;\sqrt{{\left(m_{11}\;-\;m_{22}\right)}^{2}\;+\;4m_{12}m_{21}}\right]\;.\;\;\; \end{array}$$

Due to length of explicit form of the Equation (25), the readers can find them in Appendix B. At the same time, the complete list of partially transposed density matrices ${\hat{\rho }}^{T_{1/2}}$ for the mixed spin-(1/2,*S*) Heisenberg dimers with specific spin values S=1,3/2,2,5/2 is given in Appendix C. Analysing Equation (25) in detail one identifies that only eigenvalues $\lambda_{S_{tm}^{z}}^{-}$ can be negative, and hence, the respective bipartite entanglement is in accordance to the definition (2) determined by the formula

(26) $$\begin{array}{cc} N(\rho ) & =-\sum_{S_{tm}^{z}=-S+\frac{1}{2}}^{S-\frac{1}{2}}\min\left(0,\lambda_{S_{tm}^{z}}^{-}\right). \end{array}$$

## 3. Results and Discussion

In order to minimize the huge parametric space, all further discussions will be limited to the physically most interesting case with an isotropic exchange interaction defined through the parameter Δ=1. For simplicity, the gyromagnetic factor *g* of both types of magnetic ions is set equal to two (g=2). It is worthwhile to note that other choice of the unequal *g* factor has only the quantitative, but not qualitative, impact on all obtained observations.

### 3.1. Quantum Negativity

**Figure 1:**
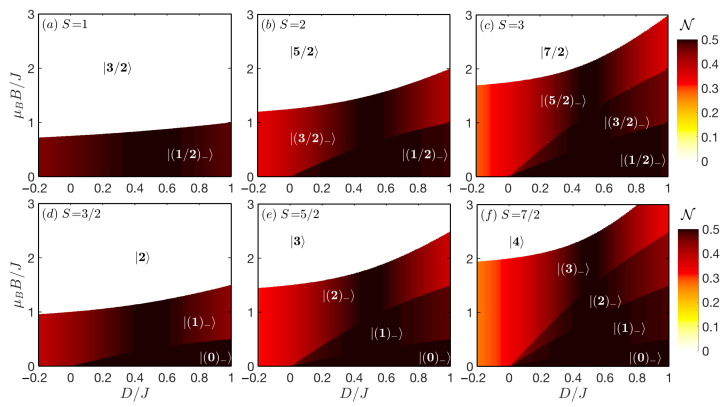
Density plots of a quantum negativity in the $D/J-\mu_{B}B/J$ plane for the spin-(1/2,*S*) Heisenberg dimers with the antiferromagnetic exchange coupling J>0 for an integer (upper panels: (**a**) S=1, (**b**) S=2, (**c**) S=3) and half-odd-integer (lower panels: (**d**) S=3/2, (**e**) S=5/2, (**f**) S=7/2) spin magnitude and isotropic interaction (Δ=1).

The behaviour of the quantum negativity of the mixed spin-(1/2,*S*) Heisenberg dimers with S=1,3/2,2,5/2,3,7/2 is illustrated in Figure 1 in the $D/J-\mu_{B}B/J$ plane by considering the antiferromagnetic exchange coupling J>0. The density plots of quantum negativity simultaneously illustrate stability regions of all relevant ground states $|{\left(S_{t}^{z}\right)}_{-}\rangle$ and |S+1/2〉.

It is worthwhile to remark that the negativity at zero magnetic field was comprehensively analysed in our preceding paper [28] and thus, the case $\mu_{B}B/J\;=\;0$ will be just marginally explored in our subsequent discussions. It has been found that the negativity at $\mu_{B}B/J\;=\;0$ exhibits qualitatively different behaviour for integer and half-odd-integer spin-*S* constituents, if the uniaxial single-ion anisotropy D/J>0 of easy-plane type is taken into account. It was surprisingly detected that the enhancement of a degree of entanglement for the mixed-spin Heisenberg dimers involving an integer spin *S* emerges as a consequence of interplay between the increasing spin magnitude *S* and uniaxial single-ion anisotropy D/J>1/2. Nevertheless, the highest negativity $N\;=\;(\sqrt{5}\;-\;1)/4$ reached for the mixed-spin Heisenberg dimers with integer spin *S* at the specific value D/J=1/2 is significantly smaller than the maximal negativity N=1/2 detected for the mixed-spin Heisenberg dimers with an arbitrary half-odd-integer spin *S*.

The external magnetic field reduces the degeneracy of the mixed spin-(1/2,*S*) Heisenberg dimers due to the Zeeman’s splitting of energy levels and the quantum negativity of an arbitrary mixed spin-(1/2,*S*) Heisenberg dimer in a state with the total spin $S_{t}^{z}$ can be expressed through the general formula

(27) $$\begin{array}{cc} \; & N\;=c_{S,S_{t}^{z}}^{+}c_{S,S_{t}^{z}}^{-}\;=\;\frac{1}{2}\sqrt{\frac{4S\left(S\;+\;1\right)\;-\;\left(2S_{t}^{z}\;-\;1\right)\left(2S_{t}^{z}\;+\;1\right)}{{\left(2S_{t}^{z}\right)}^{2}{\left(1\;-\;2\frac{D}{J}\right)}^{2}\;+\;4S\left(S\;+\;1\right)\;-\;\left(2S_{t}^{z}\;-\;1\right)\left(2S_{t}^{z}\;+\;1\right)}}. \end{array}$$

It is evident from Equation (27) and Figure 1 that the quantum negativity of each $|{\left(S_{t}^{z}\right)}_{-}\rangle$ ground state is fully independent of the external magnetic field and its magnitude decreases towards to the completely separable (N=0) ferromagnetic state |1/2+S〉 with $S_{t}^{z}\;=\;S\;+\;1/2$. However, the increasing magnetic field is responsible for existence of discontinuous changes of the quantum negativity at all field-driven magnetic phase transitions. It follows from Figure 1 that maximal bipartite entanglement is reached for the specific value of the uniaxial single-ion anisotropy D/J=1/2 (similar as in the $\mu_{B}B/J\;=\;0$ case), at which an arbitrary mixed spin-(1/2,*S*) Heisenberg dimer shows, in agreement with Equation (27), the highest quantum negativity N=1/2 until the sufficiently high magnetic field reorients both spins into its direction.

The behaviour of the quantum negativity in the regime of easy-axis uniaxial single-ion anisotropy D/J≤0 confirms previously reported findings [30,33,34] that the increasing spin magnitude *S* enlarges the stability of entangled state with respect to the magnetic field. Nevertheless, the degree of respective bipartite entanglement is gradually reduced. Contrary to this, the increasing spin magnitudes *S* induces the enhancement of a quantum negativity for an arbitrary $|{\left(S_{t}^{z}\right)}_{-}\rangle$ ($S_{t}^{z}\;\leq \;S\;-\;1/2$) ground state of the mixed spin-(1/2,*S*) Heisenberg dimers assuming easy-plane single-ion anisotropy D/J>0. In contradiction to the zero-field case, the enhancement of a negativity is observed even for 0<D/J<1/2 as a consequence of reduction of ground-state degeneracy in respective parametric space. This is a very important observation from the application perspective, because variation of a magnetic ion in the mixed spin-(1/2,*S*) Heisenberg dimers, offers a relative simple alternative how to enhance the bipartite entanglement. The origin of qualitatively different behaviour of the negativity below and above D/J=0 can be explained through the respective variation of the total spin value $S_{t}^{z}$. In the ${|\left(S\;-\;1/2\right)}_{-}\rangle$ ground state emergent in easy-axis regime D/J<0, the total spin $S_{t}^{z}\;=\;S\;-\;1/2$ is gradually enhanced with increasing spin size *S*, but the difference $4S\left(S\;+\;1\right)\;-\;\left(2S_{t}^{z}\;-\;1\right)\left(2S_{t}^{z}\;+\;1\right)$ entering into the Equation (27) remains constant, 8S. Consequently, the quantum negativity decreases as the spin *S* magnitude enlarges according to the formula

(28) $$\begin{array}{c} N\;=\;{\left[\frac{{\left(1\;-\;2\frac{D}{J}\right)}^{2}{\left(2S\;-\;1\right)}^{2}}{2S}\;+\;4\right]}^{-1/2}. \end{array}$$

Considering the fixed value of the total spin $S_{t}^{z}$ and the easy-plane regime D/J>0 the difference $4S\left(S\;+\;1\right)\;-\;\left(2S_{t}^{z}\;-\;1\right)\left(2S_{t}^{z}\;+\;1\right)$ in Equation (27) enlarges with an increasing spin size *S*, which means that the denominator in rewritten form of Equation (27)

(29) $$\begin{array}{c} N\;=\;{\left[\frac{4{\left(2S_{t}^{z}\right)}^{2}{\left(1\;-\;2\frac{D}{J}\right)}^{2}}{4S\left(S\;+\;1\right)\;-\;\left(2S_{t}^{z}\;-\;1\right)\left(2S_{t}^{z}\;+\;1\right)}\;+\;4\right]}^{-1/2}. \end{array}$$

increases and the respective quantum negativity is thus naturally enhanced. In the special case of D/J=0 the negativity is an inverse function of spin magnitude *S* and thus the quantum entanglement reduces upon strengthening of the spin size *S*.

**Figure 2:**
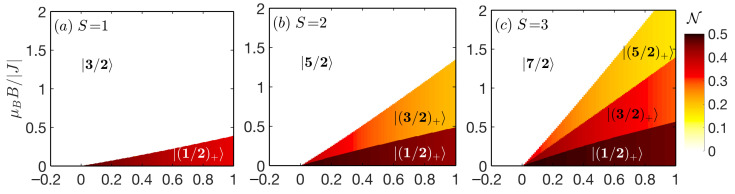

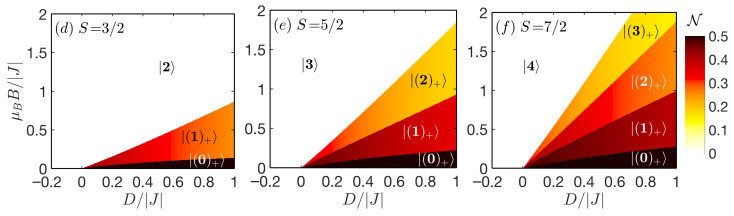
Density plots of a quantum negativity in the $D/\left|J\right|-\mu_{B}B/\left|J\right|$ plane for the spin-(1/2,*S*) Heisenberg dimers with the ferromagnetic coupling constant J<0 for an integer (upper panels: (**a**) S=1, (**b**) S=2, (**c**) S=3) and half-odd-integer (lower panels: (**d**) S=3/2, (**e**) S=5/2, (**f**) S=7/2) and isotropic interaction (Δ=1).

For the ferromagnetic exchange coupling J<0 (Figure 2), the quantum entanglement can be achieved only for the mixed-spin Heisenberg dimers with an easy-plane single-ion anisotropy D/|J|>0 due to possible preference of various ferrimagnetic or antiferromagnetic ground states $|{\left(S_{t}^{z}\right)}_{+}\rangle$ ($S_{t}^{z}\;\leq \;S\;-\;1/2$). Since the previously derived relation remains in force, the quantum negativity of each $|{\left(S_{t}^{z}\right)}_{+}\rangle$ ground state with the total spin $S_{t}^{z}\;\leq \;S\;-\;1/2$ always increases as the spin magnitudes increase. It should be pointed out that the degree of bipartite entanglement for an arbitrary $|{\left(S_{t}^{z}\right)}_{+}\rangle$ ground state is significantly smaller in comparison to its antiferromagnetic counterpart, which makes the ferromagnetic quantum mixed-spin Heisenberg dimers less attractive for practical utilizations. For a completeness, it should be emphasized, that the invariant point at D/|J|=1/2 is completely absent in the ferromagnetic case J<0 and the highest negativity (excluding the ground state ${|\left(0\right)}_{+}\rangle$) can be found in a proximity of the isotropic point D/|J|=0. Strictly at D/|J|=0 and $\mu_{B}B/J\;=\;0$ all $|{\left(S_{t}^{z}\right)}_{+}\rangle$ ($|S_{t}^{z}|\;\leq \;S\;-\;1/2$) ground states and |±(S+1/2)〉 ones are degenerate and the respective negativity follows the simple relation

(30) $$\begin{array}{c} N\;=\;\frac{\left(S\;-\;1\right)}{\left(S\;+\;1)(2S\;+\;1\right)}. \end{array}$$

### 3.2. The Thermal Negativity

**Figure 3:**
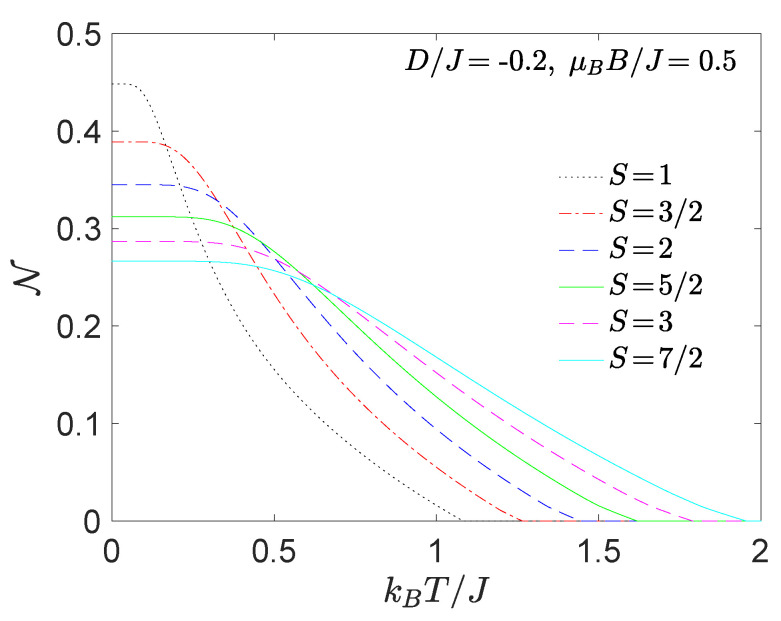
The thermal dependence of the negativity for a few different values of spin magnitudes *S* calculated at D/J=−0.2 and $\mu_{B}B/J\;=\;0.5$. The choice of model parameters corresponds to the region where the ground state ${|\left(S\;-\;1/2\right)}_{-}\rangle$ is favoured.

**Figure 4:**
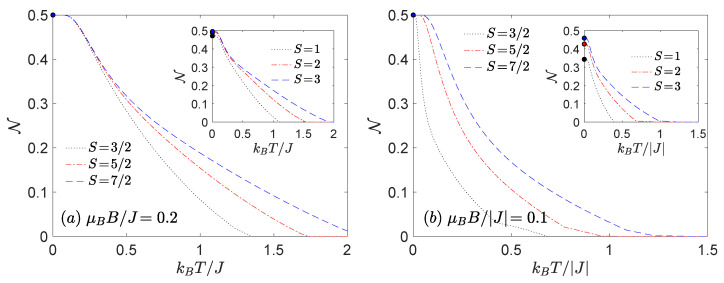
The thermal dependence of the negativity for a few different values of spin magnitudes *S* calculated at D/J=1 and (**a**) $\mu_{B}B/J\;=\;0.2$, J>0 or (**b**) $\mu_{B}B/\left|J\right|\;=\;0.1$, J<0. The choice of model parameters corresponds to the region where the ground state ${|\left(0\right)}_{\pm }\rangle$ (half-odd-integer spin *S*) or ${|\left(1/2\right)}_{\pm }\rangle$ (integer spin *S*) are favoured.

**Figure 5:**
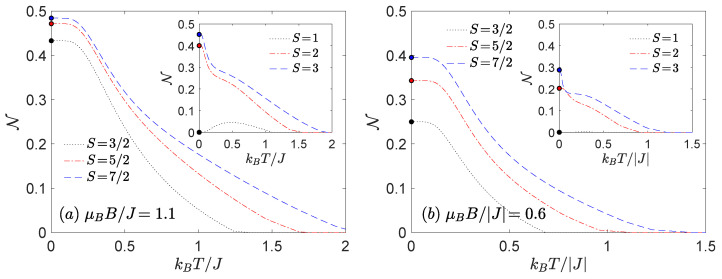
The thermal dependence of the negativity for a few different values of spin magnitudes *S* calculated at D/J=1 and (**a**) $\mu_{B}B/J\;=\;1.1$, J>0 or (**b**) $\mu_{B}B/\left|J\right|\;=\;0.6$, J<0. The choice of model parameters corresponds to the region where the ground state ${|\left(1\right)}_{\pm }\rangle$ (half-odd-integer spin *S*) or ${|\left(3/2\right)}_{\pm }\rangle$ (integer spin *S*) are favoured. In case of S=1 the |S+1/2〉 ground state is realized in both panels.

In order to analyse the thermal behaviour of the negativity we have chosen the specific sets of model parameters under the influence of magnetic field being consistent with ${|\left(S\;-\;1\right)}_{-}\rangle$ ground state (Figure 3), ${|\left(0\right)}_{\pm }\rangle$ or the ${|\left(1/2\right)}_{\pm }\rangle$ ground state (Figure 4) and finally with the ${|\left(1\right)}_{\pm }\rangle$ or ${|\left(3/2\right)}_{\pm }\rangle$ ones (Figure 5).

Focusing on Figure 3 we can generalize our previous zero-temperature conclusion [28], which states that the increasing spin magnitude *S* reduces the quantum as well as low-temperature thermal negativity. On the other hand, the increasing spin magnitude *S* enlarges the threshold temperature, which subsequently allows us to detect a subtle enhancement of the thermal entanglement at larger temperatures if the spin size *S* increases. The obtained results are in a perfect quantitative agreement with previous observations for the Heisenberg dimers without the uniaxial single-ion anisotropy D/J [26,29,33,34].

The most significant finding follows from Figure 4 and Figure 5. It is evident from these figures that the increasing spin magnitude *S* can enhance not only the threshold temperature, but it can also enhance the degree of thermal entanglement in contrast to previous knowledge. It should be emphasized that the above statement holds provided that the ensemble of integer or half-odd-integer spins *S* is taken into account separately. At low magnetic fields, the thermal negativity of Heisenberg dimers consisting of both half-odd-integer-spins always saturate in the maximal value N=1/2 in the asymptotic limit of absolute zero temperature, whereas the maximum of negativity of Heisenberg dimers composed of integer and one-half spin converges to the value $N\;=\;\frac{1}{2}\sqrt{\frac{4S(S\;+\;1)}{{\left(1\;-\;2D/J\right)}^{2}\;+\;4S\left(S\;+\;1\right)}}$. Hence, one can immediately conclude that the negativity of the Heisenberg dimers with integer spins *S* can reach the maximum value N=1/2 just for the special case D/J=1/2.

**Figure 6:**
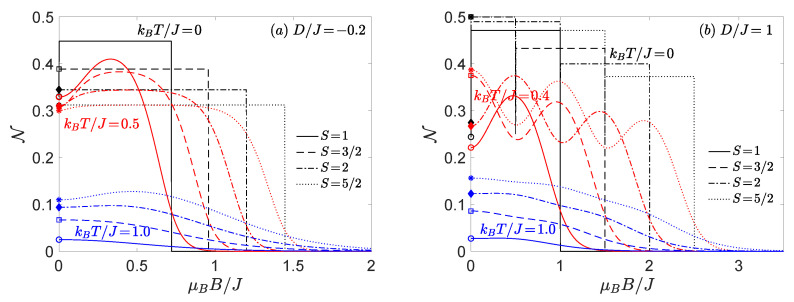
The negativity as a function of the external magnetic field for four different spin magnitudes, three different values of temperature and two selected values of the uniaxial single-ion anisotropy: (**a**) D/J=−0.2; (**b**) D/J=1.

Furthermore, in Figure 6, we present, the behaviour of the thermal negativity under the changes of magnetic field. The same model parameters have been used as in the above analysis. It should be emphasized that absence of Zeeman’s term leads to the ground-state degeneracy in the zero-temperature limit and thus, the zero-field negativity is always smaller than that in an arbitrary small but non-zero magnetic field. This fact is visualized through the symbols on the *y*-axis determining the respective asymptotic values of the negativity in zero-field limit.

In agreement with general expectation the increasing temperature reduces the bipartite entanglement with a significant drop of the negativity emergent in a proximity of all magnetic-field-induced phase transitions associated with crossing of energy levels. Around each level-crossing field the interplay between thermal and quantum fluctuations is the most pronounced and the negativity shows a marked local maximum located between two neighbouring level-crossing fields. In a consequence of that, the mixed spin-(1/2,*S*) Heisenberg dimers exhibit very specific oscillating changes of the negativity at low and moderate temperatures (Figure 6b). It is worthwhile to remark that such oscillating behaviour is possible only for the spin-(1/2,*S*) Heisenberg dimers with higher spin magnitude S>1, because existence of at least two level-crossing fields has to be guaranteed. In addition it turns out that the distance between two local maxima can be tuned through the uniaxial single-ion anisotropy D/J, which unambiguously determines the stability region (field range) of a given magnetic ground state (see Figure 1 and Figure 2).

**Figure 7:**
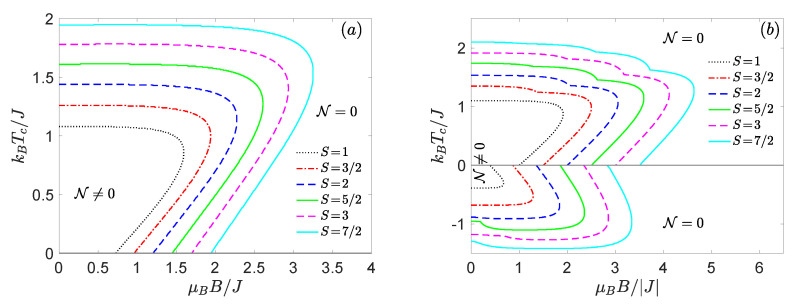
The behaviour of the threshold temperature as a function of external magnetic field and various spin-*S* magnitude. Results are calculated for two different values of uniaxial single-ion anisotropy: (**a**) D/J=−0.2 and (**b**) D/J=1, respectively.

Finally, let us turn our attention to the dependence of the threshold temperature on the spin magnitude *S* as well as the external magnetic field. The results presented in Figure 7 in the form of the threshold temperature versus magnetic field plot for a few selected spin sizes *S* confirm previous conclusions [26,29,33,34] that the threshold temperature gradually enlarges with an enhancement of the spin magnitude *S*. It is also quite evident from Figure 7b that the antiferromagnetic mixed spin-(1/2,*S*) Heisenberg dimers are more persistent against rising temperature and magnetic field than their ferromagnetic counterparts (the negative values of $k_{B}T_{c}/J$ in Figure 7b correspond to the mixed-spin Heisenberg dimers with the ferromagnetic exchange coupling J<0). Another remarkable finding is that all investigated mixed spin-(1/2,S) Heisenberg dimers exhibit a striking reentrant behaviour of the threshold temperature regardless of the character and size of the exchange coupling and the uniaxial single-ion anisotropy. The origin of the unconventional reentrant phenomenon could be explained by incapability of the magnetic field to suppress the thermally induced population of the entangled excited states, which is reflected in a thermally stimulated rise of the negativity. It appears worthwhile to remark that existence of magnetic-field-driven phase transitions gives rise to a stepwise dependence of the respective threshold temperature. The threshold temperature slightly decreases at each level-crossing field for the antiferromagnetic Heisenberg dimers (J>0), while an opposite effect is observed for the ferromagnetic Heisenberg dimers (J<0), see Figure 7b.

## 4. Conclusions

In the present paper, we have exactly examined the effect of the spin magnitude *S*, magnetic field and uniaxial single-ion anisotropy on the quantum and thermal entanglement of the mixed spin-(1/2,*S*) Heisenberg dimers. In particular, it has been verified that the concurrent interplay of the uniaxial single-ion anisotropy and the external magnetic field basically influences bipartite entanglement of the mixed-spin Heisenberg dimers. To quantify the degree of bipartite entanglement we have derived the exact analytical expression for the negativity in terms of Peres-Horodecki criterion [53,54] followed by the mathematical formulation due to Vidal and Werner [55]. In the present study, we have provided first an exhaustive analysis of all possible ground states of the mixed spin-(1/2,*S*) Heisenberg dimers as a necessarily prerequisite for further entanglement analysis. Two different scenarios were observed for both the antiferromagnetic (J>0) and ferromagnetic (J<0) coupling constants depending on the character of an uniaxial single-ion anisotropy D/J. For the easy-axis single-ion anisotropy D/J<0 the mixed-spin Heisenberg dimers exhibit either one or none magnetic-field-driven phase transition, whereas the increasing magnetic field generates S+(2Smod2)/2 consecutive field-driven phase transitions between the ground states with the total spin $\left|S_{t}^{z}\right|\leq S-1/2$ for the easy-plane single-ion anisotropy D/J>0.

As a direct consequence of different effect of easy-axis and easy-plane uniaxial single-ion anisotropy one detects very different influence of increasing spin magnitude *S* on the bipartite entanglement. In the case of easy-axis uniaxial single-ion anisotropy, an increasing spin *S* always suppresses the degree of quantum entanglement as dictated by the formula Equation (28). In contrast to this, the increasing spin magnitude *S* for the easy-plane single-ion anisotropy D/J>0 leads to the coincidence of regions with a fixed number of the total spin $S_{t}^{z}\;\leq \;S\;-\;1/2$, and in accordance with the Formula (29), the enhancement of a quantum entanglement can be observed. Interestingly, two specific conditions with maximal entanglement invariant on external stimuli have been identified for: (i) antiferromagnetic mixed spin-(1/2,*S*) Heisenberg dimers with an arbitrary spin-*S* magnitude for the particular value of the uniaxial single-ion anisotropy D/J=1/2, or (ii) the antiferromagnetic ground state ${|\left(0\right)}_{-}\rangle$ exclusively existing only in the mixed spin-(1/2,*S*) Heisenberg dimers with both half-odd-integer spin constituents.

The comprehensive analysis of a thermal entanglement above a unique ground state ${|\left(S\;-\;1/2\right)}_{-}\rangle$ confirms previously reported findings that the increasing spin magnitude *S* enlarges the threshold temperature, but unfortunately reduces the degree of thermal entanglement. However, a completely novel and unexpected behaviour has been observed for the easy-plane single-ion anisotropy D/J>0, where the increasing spin *S* may simultaneously enlarge the threshold temperature as well as the degree of thermal entanglement if one compares solely integer or half-odd-integer spin-*S* cases. It has been evidenced that except the particular case with D/J=1/2 the mixed spin-(1/2,*S*) Heisenberg dimers with both half-odd-integer spins generally achieve higher degree of entanglement, which makes them more attractive for a practical utilization. Last but not least, a fascinating oscillating changes of the negativity were observed upon the variation of the external magnetic field at low and moderate temperatures. The unconventional oscillating behaviour of the negativity originates from existence of consecutive field-driven phase transitions emergent at level-crossing fields, at which the negativity rapidly falls down before it produces significant local maxima localized in between two level-crossing fields. It is noteworthy that the local maxima of the negativity of the mixed spin-(1/2,*S*) Heisenberg dimers with integer and half-odd-integer spin *S* are not equal due to existence of different ground states in the parameter region of the easy-plane single-ion anisotropy D/J>0. Of course, the envelope of such oscillations is gradually suppressed upon strengthening of the magnetic field until the fully polarized state is reached. 

## Data Availability

The data presented in this study are available on request from the corresponding author.

## Appendix A. Density Matrices

### Appendix A.1. Density Matrix of the Mixed Spin-(1/2,1) Heisenberg Dimer

The density matrix of the mixed spin-(1/2,1) Heisenberg dimer can be recast into the following block-diagonal form

(A1) $$\begin{array}{c} \hat{\rho }\;=\;\begin{array}{cc} & \begin{array}{cccccc} |\frac{1}{2},1\rangle & |\;-\;\frac{1}{2},\;-\;1\rangle & |\frac{1}{2},0\rangle & |\;-\;\frac{1}{2},1\rangle & |\frac{1}{2},\;-\;1\rangle & |\;-\;\frac{1}{2},0\rangle \end{array}\\ \begin{array}{c} \langle \frac{1}{2},1|\;\;\;\\ \langle \;-\;\frac{1}{2},\;-\;1|\;\;\;\\ \langle \frac{1}{2},0|\;\;\;\\ \langle \;-\;\frac{1}{2},1|\;\;\;\\ \langle \frac{1}{2},\;-\;1|\;\;\;\\ \langle \;-\;\frac{1}{2},0|\;\;\; \end{array} & \left(\begin{array}{cccccc} \rho_{1,1} & \;0 & \;0 & \;0 & \;0 & \;0\\ 0 & \;\rho_{6,6} & \;0 & \;0 & \;0 & \;0\\ 0 & \;0 & \;\rho_{2,2} & \;\rho_{2,4} & \;0 & \;0\\ 0 & \;0 & \;\rho_{4,2} & \;\rho_{4,4} & \;0 & \;0\\ 0 & \;0 & \;0 & \;0 & \;\rho_{3,3} & \;\rho_{3,5}\\ 0 & \;0 & \;0 & \;0 & \;\rho_{5,3} & \;\rho_{5,5} \end{array}\right) \end{array}\;\;. \end{array}$$

The individual elements of the density matrix are defined as

(A2) $$\begin{array}{cc} \rho_{1,1}\; & =\;\langle \frac{1}{2},1|\hat{\rho }|\frac{1}{2},1\rangle \;=\;\frac{1}{Z}e^{-\beta \varepsilon_{1,\frac{3}{2}}}, \end{array}$$

(A3) $$\begin{array}{cc} \rho_{2,2}\; & =\;\langle \frac{1}{2},0|\hat{\rho }|\frac{1}{2},0\rangle \;=\;\frac{1}{Z}\left({\left(c_{1,\frac{1}{2}}^{-}\right)}^{2}e^{-\beta \varepsilon_{1,\frac{1}{2}}^{-}}\;+\;{\left(c_{1,\frac{1}{2}}^{+}\right)}^{2}e^{-\beta \varepsilon_{1,\frac{1}{2}}^{+}}\right), \end{array}$$

(A4) $$\begin{array}{cc} \rho_{3,3}\; & =\;\langle \frac{1}{2},-1|\hat{\rho }|\frac{1}{2},-1\rangle \;=\;\frac{1}{Z}\left({\left(c_{1,-\frac{1}{2}}^{-}\right)}^{2}e^{-\beta \varepsilon_{1,-\frac{1}{2}}^{-}}\;+\;{\left(c_{1,-\frac{1}{2}}^{+}\right)}^{2}e^{-\beta \varepsilon_{1,-\frac{1}{2}}^{+}}\right), \end{array}$$

(A5) $$\begin{array}{cc} \rho_{4,4}\; & =\;\langle -\frac{1}{2},1|\hat{\rho }|-\frac{1}{2},1\rangle \;=\;\frac{1}{Z}\left({\left(c_{1,\frac{1}{2}}^{+}\right)}^{2}e^{-\beta \varepsilon_{1,\frac{1}{2}}^{-}}\;+\;{\left(c_{1,\frac{1}{2}}^{-}\right)}^{2}e^{-\beta \varepsilon_{1,\frac{1}{2}}^{+}}\right), \end{array}$$

(A6) $$\begin{array}{cc} \rho_{5,5}\; & =\;\langle -\frac{1}{2},0|\hat{\rho }|-\frac{1}{2},0\rangle \;=\;\frac{1}{Z}\left({\left(c_{1,-\frac{1}{2}}^{+}\right)}^{2}e^{-\beta \varepsilon_{1,-\frac{1}{2}}^{-}}\;+\;{\left(c_{1,-\frac{1}{2}}^{-}\right)}^{2}e^{-\beta \varepsilon_{1,-\frac{1}{2}}^{+}}\right), \end{array}$$

(A7) $$\begin{array}{cc} \rho_{6,6}\; & =\;\langle -\frac{1}{2},-1|\hat{\rho }|-\frac{1}{2},-1\rangle \;=\;\frac{1}{Z}e^{-\beta \varepsilon_{1,-\frac{3}{2}}},\\ \rho_{2,4}\; & =\;\langle \frac{1}{2},0|\hat{\rho }|-\frac{1}{2},1\rangle \;=\;\frac{c_{1,\frac{1}{2}}^{+}c_{1,\frac{1}{2}}^{-}}{Z}\left(e^{-\beta \varepsilon_{1,\frac{1}{2}}^{+}}\;-\;e^{-\beta \varepsilon_{1,\frac{1}{2}}^{-}}\right), \end{array}$$

(A8) $$\begin{array}{cc} \rho_{4,2}\; & =\;\langle -\frac{1}{2},1|\hat{\rho }|\frac{1}{2},0\rangle \;=\;\frac{c_{1,\frac{1}{2}}^{+}c_{1,\frac{1}{2}}^{-}}{Z}\left(e^{-\beta \varepsilon_{1,\frac{1}{2}}^{+}}\;-\;e^{-\beta \varepsilon_{1,\frac{1}{2}}^{-}}\right).\\ \rho_{3,5}\; & =\;\langle \frac{1}{2},-1|\hat{\rho }|-\frac{1}{2},0\rangle \;=\;\frac{c_{1,-\frac{1}{2}}^{+}c_{1,-\frac{1}{2}}^{-}}{Z}\left(e^{-\beta \varepsilon_{1,-\frac{1}{2}}^{+}}\;-\;e^{-\beta \varepsilon_{1,-\frac{1}{2}}^{-}}\right), \end{array}$$

(A9) $$\begin{array}{cc} \rho_{5,3}\; & =\;\langle -\frac{1}{2},0|\hat{\rho }|\frac{1}{2},-1\rangle \;=\;\frac{c_{1,-\frac{1}{2}}^{+}c_{1,-\frac{1}{2}}^{-}}{Z}\left(e^{-\beta \varepsilon_{1,-\frac{1}{2}}^{+}}\;-\;e^{-\beta \varepsilon_{1,-\frac{1}{2}}^{-}}\right). \end{array}$$

The partition function Z of the mixed spin-(1/2,1) Heisenberg dimer reads as follows

(A10) $$\begin{array}{cc} Z & \;=\;2\left\{e^{-\frac{\beta }{2}\left(J+2D\right)}\cosh\left(\frac{3\beta h}{2}\right)\;+\;2e^{\frac{\beta }{4}\left(J-2D\right)}\cosh\left[\frac{\beta }{4}\sqrt{{\left(J\;-\;2D\right)}^{2}\;+\;8{\left(J\Delta \right)}^{2}}\right]\cosh\left(\frac{\beta h}{2}\right)\right\}. \end{array}$$

Finally, the probability coefficients $c_{S,S_{t}^{z}}^{\pm }$ and the respective energies $\varepsilon_{S,S_{t}^{z}}^{\pm }$ ($S_{t}^{z}=-3/2$, −1/2, 1/2, 3/2) have the explicit forms

(A11) $$\begin{array}{cc} c_{1,-\frac{1}{2}}^{\mp } & \;=\;\frac{1}{\sqrt{2}}\sqrt{1\;\pm \;\frac{\left(J\;-\;2D\right)}{\sqrt{{\left(J\;-\;2D\right)}^{2}\;+\;8{\left(J\Delta \right)}^{2}}}},\;\varepsilon_{1,-\frac{1}{2}}^{\mp }\;=\;-\frac{1}{4}\left(J\;-\;2D\;-\;2h\right)\;\mp \;\frac{1}{4}\sqrt{{\left(J\;-\;2D\right)}^{2}\;+\;8{\left(J\Delta \right)}^{2}}, \end{array}$$

(A12) $$\begin{array}{cc} c_{1,\frac{1}{2}}^{\mp } & \;=\;\frac{1}{\sqrt{2}}\sqrt{1\;\mp \;\frac{J\;-\;2D}{\sqrt{{\left(J\;-\;2D\right)}^{2}\;+\;8{\left(J\Delta \right)}^{2}}}},\;\varepsilon_{1,\frac{1}{2}}^{\mp }\;=\;-\frac{1}{4}\left(J\;-\;2D\;+\;2h\right)\;\mp \;\frac{1}{4}\sqrt{{\left(J\;-\;2D\right)}^{2}\;+\;8{\left(J\Delta \right)}^{2}}, \end{array}$$

(A13) $$\begin{array}{cc} c_{1,\pm \frac{3}{2}} & \;=\;1,\;\varepsilon_{1,\pm \frac{3}{2}}\;=\;\frac{1}{2}\left(J\;+\;2D\;\mp \;3h\right). \end{array}$$

### Appendix A.2. Density Matrix of the Mixed Spin-(1/2,3/2) Heisenberg Dimer

The density matrix of the mixed spin-(1/2,3/2) Heisenberg dimer can be recast into the following block-diagonal form

(A14) $$\begin{array}{c} \hat{\rho }\;=\;\begin{array}{cc} & \begin{array}{cccccccc} |\frac{1}{2},\frac{3}{2}\rangle & |\;-\;\frac{1}{2},\;-\;\frac{3}{2}\rangle & |\frac{1}{2},\frac{1}{2}\rangle & |\;-\;\frac{1}{2},\frac{3}{2}\rangle & |\frac{1}{2},\;-\;\frac{1}{2}\rangle & |\;-\;\frac{1}{2},\frac{1}{2}\rangle & |\frac{1}{2},\;-\;\frac{3}{2}\rangle & |\;-\;\frac{1}{2},\;-\;\frac{1}{2}\rangle \end{array}\\ \begin{array}{c} \langle \frac{1}{2},\frac{3}{2}|\;\;\;\\ \langle \;-\;\frac{1}{2},\;-\;\frac{3}{2}|\;\;\;\\ \langle \frac{1}{2},\frac{1}{2}|\;\;\;\\ \langle \;-\;\frac{1}{2},\frac{3}{2}|\;\;\;\\ \langle \frac{1}{2},\;-\;\frac{1}{2}|\;\;\;\\ \langle \;-\;\frac{1}{2},\frac{1}{2}|\;\;\;\\ \langle \frac{1}{2},\;-\;\frac{3}{2}|\;\;\;\\ \langle \;-\;\frac{1}{2},\;-\;\frac{1}{2}|\;\;\; \end{array} & \left(\begin{array}{cccccccc} \rho_{1,1} & \;0 & \;0 & \;0 & \;0 & \;0 & \;0 & \;0\\ 0 & \;\rho_{8,8} & \;0 & \;0 & \;0 & \;0 & \;0 & \;0\\ 0 & \;0 & \;\rho_{2,2} & \;\rho_{2,5} & \;0 & \;0 & \;0 & \;0\\ 0 & \;0 & \;\rho_{5,2} & \;\rho_{5,5} & \;0 & \;0 & \;0 & \;0\\ 0 & \;0 & \;0 & \;0 & \;\rho_{3,3} & \;\rho_{3,6} & \;0 & \;0\\ 0 & \;0 & \;0 & \;0 & \;\rho_{6,3} & \;\rho_{6,6} & \;0 & \;0\\ 0 & \;0 & \;0 & \;0 & \;0 & \;0 & \;\rho_{4,4} & \;\rho_{4,7}\\ 0 & \;0 & \;0 & \;0 & \;0 & \;0 & \;\rho_{7,4} & \;\rho_{7,7} \end{array}\right) \end{array}\;\;. \end{array}$$

The individual elements of the density matrix are defined as

(A15) $$\begin{array}{cc} \rho_{1,1}\; & =\;\langle \frac{1}{2},\frac{3}{2}|\hat{\rho }|\frac{1}{2},\frac{3}{2}\rangle \;=\;\frac{1}{Z}e^{-\beta \varepsilon_{\frac{3}{2},2}}, \end{array}$$

(A16) $$\begin{array}{cc} \rho_{2,2}\; & =\;\langle \frac{1}{2},\frac{1}{2}|\hat{\rho }|\frac{1}{2},\frac{1}{2}\rangle \;=\;\frac{1}{Z}\left({\left(c_{\frac{3}{2},1}^{-}\right)}^{2}e^{-\beta \varepsilon_{\frac{3}{2},1}^{-}}\;+\;{\left(c_{\frac{3}{2},1}^{+}\right)}^{2}e^{-\beta \varepsilon_{\frac{3}{2},1}^{+}}\right), \end{array}$$

(A17) $$\begin{array}{cc} \rho_{3,3}\; & =\;\langle \frac{1}{2},-\frac{1}{2}|\hat{\rho }|\frac{1}{2},-\frac{1}{2}\rangle \;=\;\frac{1}{Z}\left({\left(c_{\frac{3}{2},0}^{-}\right)}^{2}e^{-\beta \varepsilon_{\frac{3}{2},0}^{-}}\;+\;{\left(c_{\frac{3}{2},0}^{+}\right)}^{2}e^{-\beta \varepsilon_{\frac{3}{2},0}^{+}}\right), \end{array}$$

(A18) $$\begin{array}{cc} \rho_{4,4}\; & =\;\langle \frac{1}{2},-\frac{3}{2}|\hat{\rho }|\frac{1}{2},-\frac{3}{2}\rangle \;=\;\frac{1}{Z}\left({\left(c_{\frac{3}{2},-1}^{-}\right)}^{2}e^{-\beta \varepsilon_{\frac{3}{2},-1}^{-}}\;+\;{\left(c_{\frac{3}{2},-1}^{+}\right)}^{2}e^{-\beta \varepsilon_{\frac{3}{2},-1}^{+}}\right), \end{array}$$

(A19) $$\begin{array}{cc} \rho_{5,5}\; & =\;\langle -\frac{1}{2},\frac{3}{2}|\hat{\rho }|-\frac{1}{2},\frac{3}{2}\rangle \;=\;\frac{1}{Z}\left({\left(c_{\frac{3}{2},1}^{+}\right)}^{2}e^{-\beta \varepsilon_{\frac{3}{2},1}^{-}}\;+\;{\left(c_{\frac{3}{2},1}^{-}\right)}^{2}e^{-\beta \varepsilon_{\frac{3}{2},1}^{+}}\right), \end{array}$$

(A20) $$\begin{array}{cc} \rho_{6,6}\; & =\;\langle -\frac{1}{2},\frac{1}{2}|\hat{\rho }|-\frac{1}{2},\frac{1}{2}\rangle \;=\;\frac{1}{Z}\left({\left(c_{\frac{3}{2},0}^{+}\right)}^{2}e^{-\beta \varepsilon_{\frac{3}{2},0}^{-}}\;+\;{\left(c_{\frac{3}{2},0}^{-}\right)}^{2}e^{-\beta \varepsilon_{\frac{3}{2},0}^{+}}\right), \end{array}$$

(A21) $$\begin{array}{cc} \rho_{7,7}\; & =\;\langle -\frac{1}{2},-\frac{1}{2}|\hat{\rho }|-\frac{1}{2},-\frac{1}{2}\rangle \;=\;\frac{1}{Z}\left({\left(c_{\frac{3}{2},-1}^{+}\right)}^{2}e^{-\beta \varepsilon_{\frac{3}{2},-1}^{-}}\;+\;{\left(c_{\frac{3}{2},-1}^{-}\right)}^{2}e^{-\beta \varepsilon_{\frac{3}{2},-1}^{+}}\right), \end{array}$$

(A22) $$\begin{array}{cc} \rho_{8,8}\; & =\;\langle -\frac{1}{2},-\frac{3}{2}|\hat{\rho }|-\frac{1}{2},-\frac{3}{2}\rangle \;=\;\frac{1}{Z}e^{-\beta \varepsilon_{\frac{3}{2},-2}},\\ \rho_{2,5}\; & =\;\langle \frac{1}{2},\frac{1}{2}|\hat{\rho }|-\frac{1}{2},\frac{3}{2}\rangle \;=\;\frac{c_{\frac{3}{2},1}^{+}c_{\frac{3}{2},1}^{-}}{Z}\left(e^{-\beta \varepsilon_{\frac{3}{2},1}^{+}}\;-\;e^{-\beta \varepsilon_{\frac{3}{2},1}^{-}}\right), \end{array}$$

(A23) $$\begin{array}{cc} \rho_{5,2}\; & =\;\langle -\frac{1}{2},\frac{3}{2}|\hat{\rho }|\frac{1}{2},\frac{1}{2}\rangle \;=\;\frac{c_{\frac{3}{2},1}^{+}c_{\frac{3}{2},1}^{-}}{Z}\left(e^{-\beta \varepsilon_{\frac{3}{2},1}^{+}}\;-\;e^{-\beta \varepsilon_{\frac{3}{2},1}^{-}}\right),\\ \rho_{3,6}\; & =\;\langle \frac{1}{2},-\frac{1}{2}|\hat{\rho }|-\frac{1}{2},\frac{1}{2}\rangle \;=\;\frac{c_{\frac{3}{2},0}^{+}c_{\frac{3}{2},0}^{-}}{Z}\left(e^{-\beta \varepsilon_{\frac{3}{2},0}^{+}}\;-\;e^{-\beta \varepsilon_{\frac{3}{2},0}^{-}}\right), \end{array}$$

(A24) $$\begin{array}{cc} \rho_{6,3}\; & =\;\langle -\frac{1}{2},\frac{1}{2}|\hat{\rho }|\frac{1}{2},-\frac{1}{2}\rangle \;=\;\frac{c_{\frac{3}{2},0}^{+}c_{\frac{3}{2},0}^{-}}{Z}\left(e^{-\beta \varepsilon_{\frac{3}{2},0}^{+}}\;-\;e^{-\beta \varepsilon_{\frac{3}{2},0}^{-}}\right),\\ \rho_{4,7}\; & =\;\langle \frac{1}{2},-\frac{3}{2}|\hat{\rho }|-\frac{1}{2},-\frac{1}{2}\rangle \;=\;\frac{c_{\frac{3}{2},-1}^{+}c_{\frac{3}{2},-1}^{-}}{Z}\left(e^{-\beta \varepsilon_{\frac{3}{2},-1}^{+}}\;-\;e^{-\beta \varepsilon_{\frac{3}{2},-1}^{-}}\right), \end{array}$$

(A25) $$\begin{array}{cc} \rho_{7,4}\; & =\;\langle -\frac{1}{2},-\frac{1}{2}|\hat{\rho }|\frac{1}{2},-\frac{3}{2}\rangle \;=\;\frac{c_{\frac{3}{2},-1}^{+}c_{\frac{3}{2},-1}^{-}}{Z}\left(e^{-\beta \varepsilon_{\frac{3}{2},-1}^{+}}\;-\;e^{-\beta \varepsilon_{\frac{3}{2},-1}^{-}}\right). \end{array}$$

The partition function Z of the mixed spin-(1/2,3/2) Heisenberg dimer reads as follows

(A26) $$\begin{array}{cc} Z\;=\;2 & \left\{e^{-\frac{3\beta }{4}\left(J+3D\right)}\cosh\left(2\beta h\right)\;+\;2e^{\frac{\beta }{4}\left(J-5D\right)}\cosh\left(\frac{\beta }{2}\sqrt{{\left(J\;-\;2D\right)}^{2}\;+\;3{\left(J\Delta \right)}^{2}}\right)\cosh\left(\beta h\right)\right.\\ \; & \;+\;\left.e^{\frac{\beta }{4}\left(J-D\right)}\cosh\left(\beta \sqrt{{\left(J\Delta \right)}^{2}}\right)\right\}. \end{array}$$

Finally, the probability coefficients $c_{S,S_{t}^{z}}^{\pm }$ and the respective energies $\varepsilon_{S,S_{t}^{z}}^{\pm }$ ($S_{t}^{z}=-2$, −1, 0, 1, 2) have the explicit forms

(A27) $$\begin{array}{cc} c_{\frac{3}{2},-1}^{\mp } & \;=\;\frac{1}{\sqrt{2}}\sqrt{1\;\pm \;\frac{\left(J\;-\;2D\right)}{\sqrt{{\left(J\;-\;2D\right)}^{2}\;+\;3{\left(J\Delta \right)}^{2}}}},\;\varepsilon_{\frac{3}{2},-1}^{\mp }\;=\;-\frac{1}{4}\left(J\;-\;5D\;-\;4h\right)\;\mp \;\frac{1}{2}\sqrt{{\left(J\;-\;2D\right)}^{2}\;+\;3{\left(J\Delta \right)}^{2}}, \end{array}$$

(A28) $$\begin{array}{cc} c_{\frac{3}{2},0}^{\mp } & \;=\;\frac{1}{\sqrt{2}},\;\varepsilon_{\frac{3}{2},0}^{\mp }\;=\;-\frac{1}{4}\left(J\;-\;D\right)\;\mp \;\sqrt{{\left(J\Delta \right)}^{2}}, \end{array}$$

(A29) $$\begin{array}{cc} c_{\frac{3}{2},1}^{\mp } & \;=\;\frac{1}{\sqrt{2}}\sqrt{1\;\mp \;\frac{\left(J\;-\;2D\right)}{\sqrt{{\left(J\;-\;2D\right)}^{2}\;+\;3{\left(J\Delta \right)}^{2}}}},\;\varepsilon_{\frac{3}{2},1}^{\mp }\;=\;-\frac{1}{4}\left(J\;-\;5D\;+\;4h\right)\;\mp \;\frac{1}{2}\sqrt{{\left(J\;-\;2D\right)}^{2}\;+\;3{\left(J\Delta \right)}^{2}}, \end{array}$$

(A30) $$\begin{array}{cc} c_{\frac{3}{2},\pm 2} & \;=\;1,\;\varepsilon_{\frac{3}{2},\pm 2}\;=\;\frac{3}{4}\left(J\;+\;3D\;\mp \;2h\right). \end{array}$$

### Appendix A.3. Density Matrix of the Mixed Spin-(1/2,2) Heisenberg Dimer

The density matrix of the mixed spin-(1/2,2) Heisenberg dimer can be recast into the following block-diagonal form

(A31) $$\begin{array}{c} \hat{\rho }\;=\;\begin{array}{cc} & \begin{array}{cccccccccc} |\frac{1}{2},2\rangle & |\;-\;\frac{1}{2},\;-\;2\rangle & |\frac{1}{2},1\rangle & |\;-\;\frac{1}{2},2\rangle & |\frac{1}{2},0\rangle & |\;-\;\frac{1}{2},1\rangle & |\frac{1}{2},\;-\;1\rangle & |\;-\;\frac{1}{2},0\rangle & |\frac{1}{2},\;-\;2\rangle & |\;-\;\frac{1}{2},\;-\;1\rangle \end{array}\\ \begin{array}{c} \langle \frac{1}{2},2|\;\;\;\\ \langle \;-\;\frac{1}{2},\;-\;2|\;\;\;\\ \langle \frac{1}{2},1|\;\;\;\\ \langle \;-\;\frac{1}{2},2|\;\;\;\\ \langle \frac{1}{2},0|\;\;\;\\ \langle \;-\;\frac{1}{2},1|\;\;\;\\ \langle \frac{1}{2},\;-\;1|\;\;\;\\ \langle \;-\;\frac{1}{2},0|\;\;\;\\ \langle \frac{1}{2},\;-\;2|\;\;\;\\ \langle \;-\;\frac{1}{2},\;-\;1|\;\;\; \end{array} & \left(\begin{array}{c} \begin{array}{cccccccccc} \rho_{1,1} & \;0 & \;0 & \;0 & \;0 & \;0 & \;0 & \;0 & \;0 & \;0\\ 0 & \;\rho_{10,10} & \;0 & \;0 & \;0 & \;0 & \;0 & \;0 & \;0 & \;0\\ 0 & \;0 & \;\rho_{2,2} & \;\rho_{2,6} & \;0 & \;0 & \;0 & \;0 & \;0 & \;0\\ 0 & \;0 & \;\rho_{6,2} & \;\rho_{6,6} & \;0 & \;0 & \;0 & \;0 & \;0 & \;0\\ 0 & \;0 & \;0 & \;0 & \;\rho_{3,3} & \;\rho_{3,7} & \;0 & \;0 & \;0 & \;0\\ 0 & \;0 & \;0 & \;0 & \;\rho_{7,3} & \;\rho_{7,7} & \;0 & \;0 & \;0 & \;0\\ 0 & \;0 & \;0 & \;0 & \;0 & \;0 & \;\rho_{4,4} & \;\rho_{4,8} & \;0 & \;0\\ 0 & \;0 & \;0 & \;0 & \;0 & \;0 & \;\rho_{8,4} & \;\rho_{8,8} & \;0 & \;0\\ 0 & \;0 & \;0 & \;0 & \;0 & \;0 & \;0 & \;0 & \;\rho_{5,5} & \;\rho_{5,9}\\ 0 & \;0 & \;0 & \;0 & \;0 & \;0 & \;0 & \;0 & \;\rho_{9,5} & \;\rho_{9,9} \end{array} \end{array}\right) \end{array}\;\;. \end{array}$$

The individual elements of the density matrix are defined as

(A32) $$\begin{array}{cc} \rho_{1,1}\; & =\;\langle \frac{1}{2},2|\hat{\rho }|\frac{1}{2},2\rangle \;=\;\frac{1}{Z}e^{-\beta \varepsilon_{2,\frac{5}{2}}}, \end{array}$$

(A33) $$\begin{array}{cc} \rho_{2,2}\; & =\;\langle \frac{1}{2},1|\hat{\rho }|\frac{1}{2},1\rangle \;=\;\frac{1}{Z}\left({\left(c_{2,\frac{3}{2}}^{-}\right)}^{2}e^{-\beta \varepsilon_{2,\frac{3}{2}}^{-}}\;+\;{\left(c_{2,\frac{3}{2}}^{+}\right)}^{2}e^{-\beta \varepsilon_{2,\frac{3}{2}}^{+}}\right), \end{array}$$

(A34) $$\begin{array}{cc} \rho_{3,3}\; & =\;\langle \frac{1}{2},0|\hat{\rho }|\frac{1}{2},0\rangle \;=\;\frac{1}{Z}\left({\left(c_{2,\frac{1}{2}}^{-}\right)}^{2}e^{-\beta \varepsilon_{2,\frac{1}{2}}^{-}}\;+\;{\left(c_{2,\frac{1}{2}}^{+}\right)}^{2}e^{-\beta \varepsilon_{2,\frac{1}{2}}^{+}}\right), \end{array}$$

(A35) $$\begin{array}{cc} \rho_{4,4}\; & =\;\langle \frac{1}{2},-1|\hat{\rho }|\frac{1}{2},-1\rangle \;=\;\frac{1}{Z}\left({\left(c_{2,-\frac{1}{2}}^{-}\right)}^{2}e^{-\beta \varepsilon_{2,-\frac{1}{2}}^{-}}\;+\;{\left(c_{2,-\frac{1}{2}}^{+}\right)}^{2}e^{-\beta \varepsilon_{2,-\frac{1}{2}}^{+}}\right), \end{array}$$

(A36) $$\begin{array}{cc} \rho_{5,5}\; & =\;\langle \frac{1}{2},-2|\hat{\rho }|\frac{1}{2},-2\rangle \;=\;\frac{1}{Z}\left({\left(c_{2,-\frac{3}{2}}^{-}\right)}^{2}e^{-\beta \varepsilon_{2,-\frac{3}{2}}^{-}}\;+\;{\left(c_{2,-\frac{3}{2}}^{+}\right)}^{2}e^{-\beta \varepsilon_{2,-\frac{3}{2}}^{+}}\right), \end{array}$$

(A37) $$\begin{array}{cc} \rho_{6,6}\; & =\;\langle -\frac{1}{2},2|\hat{\rho }|-\frac{1}{2},2\rangle \;=\;\frac{1}{Z}\left({\left(c_{2,\frac{3}{2}}^{+}\right)}^{2}e^{-\beta \varepsilon_{2,\frac{3}{2}}^{-}}\;+\;{\left(c_{2,\frac{3}{2}}^{-}\right)}^{2}e^{-\beta \varepsilon_{2,\frac{3}{2}}^{+}}\right), \end{array}$$

(A38) $$\begin{array}{cc} \rho_{7,7}\; & =\;\langle -\frac{1}{2},1|\hat{\rho }|-\frac{1}{2},1\rangle \;=\;\frac{1}{Z}\left({\left(c_{2,\frac{1}{2}}^{+}\right)}^{2}e^{-\beta \varepsilon_{2,\frac{1}{2}}^{-}}\;+\;{\left(c_{2,\frac{1}{2}}^{-}\right)}^{2}e^{-\beta \varepsilon_{2,\frac{1}{2}}^{+}}\right), \end{array}$$

(A39) $$\begin{array}{cc} \rho_{8,8}\; & =\;\langle -\frac{1}{2},0|\hat{\rho }|-\frac{1}{2},0\rangle \;=\;\frac{1}{Z}\left({\left(c_{2,-\frac{1}{2}}^{+}\right)}^{2}e^{-\beta \varepsilon_{2,-\frac{1}{2}}^{-}}\;+\;{\left(c_{2,-\frac{1}{2}}^{-}\right)}^{2}e^{-\beta \varepsilon_{2,-\frac{1}{2}}^{+}}\right), \end{array}$$

(A40) $$\begin{array}{cc} \rho_{9,9}\; & =\;\langle -\frac{1}{2},-1|\hat{\rho }|-\frac{1}{2},-1\rangle \;=\;\frac{1}{Z}\left({\left(c_{2,-\frac{3}{2}}^{+}\right)}^{2}e^{-\beta \varepsilon_{2,-\frac{3}{2}}^{-}}\;+\;{\left(c_{2,-\frac{3}{2}}^{-}\right)}^{2}e^{-\beta \varepsilon_{2,-\frac{3}{2}}^{+}}\right), \end{array}$$

(A41) $$\begin{array}{cc} \rho_{10,10}\; & =\;\langle -\frac{1}{2},-2|\hat{\rho }|-\frac{1}{2},-2\rangle \;=\;\frac{1}{Z}e^{-\beta \varepsilon_{2,-\frac{5}{2}}},\\ \rho_{2,6}\; & =\;\langle \frac{1}{2},1|\hat{\rho }|-\frac{1}{2},2\rangle \;=\;\frac{c_{2,\frac{3}{2}}^{+}c_{2,\frac{3}{2}}^{-}}{Z}\left(e^{-\beta \varepsilon_{2,\frac{3}{2}}^{+}}\;-\;e^{-\beta \varepsilon_{2,\frac{3}{2}}^{-}}\right), \end{array}$$

(A42) $$\begin{array}{cc} \rho_{6,2}\; & =\;\langle -\frac{1}{2},2|\hat{\rho }|\frac{1}{2},1\rangle \;=\;\frac{c_{2,\frac{3}{2}}^{+}c_{2,\frac{3}{2}}^{-}}{Z}\left(e^{-\beta \varepsilon_{2,\frac{3}{2}}^{+}}\;-\;e^{-\beta \varepsilon_{2,\frac{3}{2}}^{-}}\right),\\ \rho_{3,7}\; & =\;\langle \frac{1}{2},0|\hat{\rho }|-\frac{1}{2},1\rangle \;=\;\frac{c_{2,\frac{1}{2}}^{+}c_{2,\frac{1}{2}}^{-}}{Z}\left(e^{-\beta \varepsilon_{2,\frac{1}{2}}^{+}}\;-\;e^{-\beta \varepsilon_{2,\frac{1}{2}}^{-}}\right), \end{array}$$

(A43) $$\begin{array}{cc} \rho_{7,3}\; & =\;\langle -\frac{1}{2},1|\hat{\rho }|\frac{1}{2},0\rangle \;=\;\frac{c_{2,\frac{1}{2}}^{+}c_{2,\frac{1}{2}}^{-}}{Z}\left(e^{-\beta \varepsilon_{2,\frac{1}{2}}^{+}}\;-\;e^{-\beta \varepsilon_{2,\frac{1}{2}}^{-}}\right),\\ \rho_{4,8}\; & =\;\langle \frac{1}{2},-1|\hat{\rho }|-\frac{1}{2},0\rangle \;=\;\frac{c_{2,-\frac{1}{2}}^{+}c_{2,-\frac{1}{2}}^{-}}{Z}\left(e^{-\beta \varepsilon_{2,-\frac{1}{2}}^{+}}\;-\;e^{-\beta \varepsilon_{2,-\frac{1}{2}}^{-}}\right), \end{array}$$

(A44) $$\begin{array}{cc} \rho_{8,4}\; & =\;\langle -\frac{1}{2},0|\hat{\rho }|\frac{1}{2},-1\rangle \;=\;\frac{c_{2,-\frac{1}{2}}^{+}c_{2,-\frac{1}{2}}^{-}}{Z}\left(e^{-\beta \varepsilon_{2,-\frac{1}{2}}^{+}}\;-\;e^{-\beta \varepsilon_{2,-\frac{1}{2}}^{-}}\right),\\ \rho_{5,9}\; & =\;\langle \frac{1}{2},-2|\hat{\rho }|-\frac{1}{2},-1\rangle \;=\;\frac{c_{2,-\frac{3}{2}}^{+}c_{2,-\frac{3}{2}}^{-}}{Z}\left(e^{-\beta \varepsilon_{2,-\frac{3}{2}}^{+}}\;-\;e^{-\beta \varepsilon_{2,-\frac{3}{2}}^{-}}\right), \end{array}$$

(A45) $$\begin{array}{cc} \rho_{9,5}\; & =\;\langle -\frac{1}{2},-1|\hat{\rho }|\frac{1}{2},-2\rangle \;=\;\frac{c_{2,-\frac{3}{2}}^{+}c_{2,-\frac{3}{2}}^{-}}{Z}\left(e^{-\beta \varepsilon_{2,-\frac{3}{2}}^{+}}\;-\;e^{-\beta \varepsilon_{2,-\frac{3}{2}}^{-}}\right). \end{array}$$

The partition function Z of the mixed spin-(1/2,2) Heisenberg dimer reads as follows

(A46) $$\begin{array}{cc} Z\;=\;2 & \left\{e^{-\beta (J+4D)}\cosh\left(\frac{5\beta h}{2}\right)\;+\;2e^{\frac{\beta }{4}\left(J\;-\;10D\right)}\cosh\left(\frac{\beta }{4}\sqrt{9{\left(J\;-\;2D\right)}^{2}\;+\;16{\left(J\Delta \right)}^{2}}\right)\cosh\left(\frac{3\beta h}{2}\right)\right.\\ \; & \;+\;\left.2e^{\frac{\beta }{4}\left(J\;-\;2D\right)}\cosh\left(\frac{\beta }{4}\sqrt{{\left(J\;-\;2D\right)}^{2}\;+\;24{\left(J\Delta \right)}^{2}}\right)\cosh\left(\frac{\beta h}{2}\right)\right\}. \end{array}$$

Finally, the probability coefficients $c_{S,S_{t}^{z}}^{\pm }$ and the respective energies $\varepsilon_{S,S_{t}^{z}}^{\pm }$ ($S_{t}^{z}=-5/2$, −3/2, −1/2, 1/2, 3/2, 5/2) have the explicit forms

(A47) $$\begin{array}{cc} c_{2,-\frac{3}{2}}^{\mp } & \;=\;\frac{1}{\sqrt{2}}\sqrt{1\;\pm \;\frac{3(J\;-\;2D)}{\sqrt{9{\left(J\;-\;2D\right)}^{2}\;+\;16{\left(J\Delta \right)}^{2}}}},\;\varepsilon_{2,-\frac{3}{2}}^{\mp }\;=\;-\frac{1}{4}\left(J\;-\;10D\;-\;6h\right)\;\mp \;\frac{1}{4}\sqrt{9{\left(J\;-\;2D\right)}^{2}\;+\;16{\left(J\Delta \right)}^{2}}, \end{array}$$

(A48) $$\begin{array}{cc} c_{2,-\frac{1}{2}}^{\mp } & \;=\;\frac{1}{\sqrt{2}}\sqrt{1\;\pm \;\frac{\left(J\;-\;2D\right)}{\sqrt{{\left(J\;-\;2D\right)}^{2}\;+\;24{\left(J\Delta \right)}^{2}}}},\;\;\varepsilon_{2,-\frac{1}{2}}^{\mp }\;=\;-\frac{1}{4}\left(J\;-\;2D\;-\;2h\right)\;\mp \;\frac{1}{4}\sqrt{{\left(J\;-\;2D\right)}^{2}\;+\;24{\left(J\Delta \right)}^{2}}, \end{array}$$

(A49) $$\begin{array}{cc} c_{2,\frac{1}{2}}^{\mp } & \;=\;\frac{1}{\sqrt{2}}\sqrt{1\;\mp \;\frac{\left(J\;-\;2D\right)}{\sqrt{{\left(J\;-\;2D\right)}^{2}\;+\;24{\left(J\Delta \right)}^{2}}}},\;\;\varepsilon_{2,\frac{1}{2}}^{\mp }\;=\;-\frac{1}{4}\left(J\;-\;2D\;+\;2h\right)\;\mp \;\frac{1}{4}\sqrt{{\left(J\;-\;2D\right)}^{2}\;+\;24{\left(J\Delta \right)}^{2}}, \end{array}$$

(A50) $$\begin{array}{cc} c_{2,\frac{3}{2}}^{\mp } & \;=\;\frac{1}{\sqrt{2}}\sqrt{1\;\mp \;\frac{3(J\;-\;2D)}{\sqrt{9{\left(J\;-\;2D\right)}^{2}\;+\;16{\left(J\Delta \right)}^{2}}}},\;\varepsilon_{2,\frac{3}{2}}^{\mp }\;=\;-\frac{1}{4}\left(J\;-\;10D\;+\;6h\right)\;\mp \;\frac{1}{4}\sqrt{9{\left(J\;-\;2D\right)}^{2}\;+\;16{\left(J\Delta \right)}^{2}}, \end{array}$$

(A51) $$\begin{array}{cc} c_{2,\pm \frac{5}{2}} & \;=\;1,\;\varepsilon_{2,\pm \frac{5}{2}}\;=\;J\;+\;4D\;\mp \;\frac{5h}{2}. \end{array}$$

### Appendix A.4. Density Matrix of the Mixed Spin-(1/2,5/2) Heisenberg Dimer

The density matrix of the mixed spin-(1/2,5/2) Heisenberg dimer can be recast into the following block-diagonal form

(A52) $$\begin{array}{c} \hat{\rho }\;=\;\begin{array}{cc} & \begin{array}{cccccccccccc} |\frac{1}{2},\frac{5}{2}\rangle & |\;-\;\frac{1}{2},\;-\;\frac{5}{2}\rangle & |\frac{1}{2},\frac{3}{2}\rangle & |\;-\;\frac{1}{2},\frac{5}{2}\rangle & |\frac{1}{2},\frac{1}{2}\rangle & |\;-\;\frac{1}{2},\frac{3}{2}\rangle & |\frac{1}{2},\;-\;\frac{1}{2}\rangle & |\;-\;\frac{1}{2},\;-\;\frac{1}{2}\rangle & |\frac{1}{2},\;-\;\frac{3}{2}\rangle & |\;-\;\frac{1}{2},\;-\;\frac{1}{2}\rangle & |\frac{1}{2},\;-\;\frac{5}{2}\rangle & |\;-\;\frac{1}{2},\;-\;\frac{3}{2}\rangle \end{array}\\ \begin{array}{c} \langle \frac{1}{2},\frac{1}{2}|\;\;\;\\ \langle \;-\;\frac{1}{2},\;-\;\frac{5}{2}|\;\;\;\\ \langle \frac{1}{2},\frac{3}{2}|\;\;\;\\ \langle \;-\;\frac{1}{2},\frac{5}{2}|\;\;\;\\ \langle \frac{1}{2},\frac{1}{2}|\;\;\;\\ \langle \;-\;\frac{1}{2},\frac{3}{2}|\;\;\;\\ \langle \frac{1}{2},\;-\;\frac{1}{2}|\;\;\;\\ \langle \;-\;\frac{1}{2},\;-\;\frac{1}{2}|\;\;\;\\ \langle \frac{1}{2},\;-\;\frac{3}{2}|\;\;\;\\ \langle \;-\;\frac{1}{2},\;-\;\frac{1}{2}|\;\;\;\\ \langle \frac{1}{2},\;-\;\frac{5}{2}|\;\;\;\\ \langle \;-\;\frac{1}{2},\;-\;\frac{3}{2}|\;\;\; \end{array} & \left(\begin{array}{c} \begin{array}{cccccccccccc} \rho_{1,1} & \;0 & \;0 & \;0 & \;0 & \;0 & \;0 & \;0 & \;0 & \;0 & \;0 & \;0\\ 0 & \;\rho_{12,12} & \;0 & \;0 & \;0 & \;0 & \;0 & \;0 & \;0 & \;0 & \;0 & \;0\\ 0 & \;0 & \;\rho_{2,2} & \;\rho_{2,7} & \;0 & \;0 & \;0 & \;0 & \;0 & \;0 & \;0 & \;0\\ 0 & \;0 & \;\rho_{7,2} & \;\rho_{7,7} & \;0 & \;0 & \;0 & \;0 & \;0 & \;0 & \;0 & \;0\\ 0 & \;0 & \;0 & \;0 & \;\rho_{3,3} & \;\rho_{3,8} & \;0 & \;0 & \;0 & \;0 & \;0 & \;0\\ 0 & \;0 & \;0 & \;0 & \;\rho_{8,3} & \;\rho_{8,8} & \;0 & \;0 & \;0 & \;0 & \;0 & \;0\\ 0 & \;0 & \;0 & \;0 & \;0 & \;0 & \;\rho_{4,4} & \;\rho_{4,9} & \;0 & \;0 & \;0 & \;0\\ 0 & \;0 & \;0 & \;0 & \;0 & \;0 & \;\rho_{9,4} & \;\rho_{9,9} & \;0 & \;0 & \;0 & \;0\\ 0 & \;0 & \;0 & \;0 & \;0 & \;0 & \;0 & \;0 & \;\rho_{5,5} & \;\rho_{5,10} & \;0 & \;0\\ 0 & \;0 & \;0 & \;0 & \;0 & \;0 & \;0 & \;0 & \;\rho_{10,5} & \;\rho_{10,10} & \;0 & \;0\\ 0 & \;0 & \;0 & \;0 & \;0 & \;0 & \;0 & \;0 & \;0 & \;0 & \;\rho_{6,6} & \;\rho_{6,11}\\ 0 & \;0 & \;0 & \;0 & \;0 & \;0 & \;0 & \;0 & \;0 & \;0 & \;\rho_{11,6} & \;\rho_{11,11} \end{array} \end{array}\right) \end{array}\;\;. \end{array}$$

The individual elements of the density matrix are defined as

(A53) $$\begin{array}{cc} \rho_{1,1}\; & =\;\langle \frac{1}{2},\frac{5}{2}|\hat{\rho }|\frac{1}{2},\frac{5}{2}\rangle \;=\;\frac{1}{Z}e^{-\beta \varepsilon_{\frac{5}{2},3}}, \end{array}$$

(A54) $$\begin{array}{cc} \rho_{2,2}\; & =\;\langle \frac{1}{2},\frac{3}{2}|\hat{\rho }|\frac{1}{2},\frac{3}{2}\rangle \;=\;\frac{1}{Z}\left({\left(c_{\frac{5}{2},2}^{-}\right)}^{2}e^{-\beta \varepsilon_{\frac{5}{2},2}^{-}}\;+\;{\left(c_{\frac{5}{2},2}^{+}\right)}^{2}e^{-\beta \varepsilon_{\frac{5}{2},2}^{+}}\right), \end{array}$$

(A55) $$\begin{array}{cc} \rho_{3,3}\; & =\;\langle \frac{1}{2},\frac{1}{2}|\hat{\rho }|\frac{1}{2},\frac{1}{2}\rangle \;=\;\frac{1}{Z}\left({\left(c_{\frac{5}{2},1}^{-}\right)}^{2}e^{-\beta \varepsilon_{\frac{5}{2},1}^{-}}\;+\;{\left(c_{\frac{5}{2},1}^{+}\right)}^{2}e^{-\beta \varepsilon_{\frac{5}{2},1}^{+}}\right), \end{array}$$

(A56) $$\begin{array}{cc} \rho_{4,4}\; & =\;\langle \frac{1}{2},-\frac{1}{2}|\hat{\rho }|\frac{1}{2},-\frac{1}{2}\rangle \;=\;\frac{1}{Z}\left({\left(c_{\frac{5}{2},0}^{-}\right)}^{2}e^{-\beta \varepsilon_{\frac{5}{2},0}^{-}}\;+\;{\left(c_{\frac{5}{2},0}^{+}\right)}^{2}e^{-\beta \varepsilon_{\frac{5}{2},0}^{+}}\right), \end{array}$$

(A57) $$\begin{array}{cc} \rho_{5,5}\; & =\;\langle \frac{1}{2},-\frac{3}{2}|\hat{\rho }|\frac{1}{2},-\frac{3}{2}\rangle \;=\;\frac{1}{Z}\left({\left(c_{\frac{5}{2},-1}^{-}\right)}^{2}e^{-\beta \varepsilon_{\frac{5}{2},-1}^{-}}\;+\;{\left(c_{\frac{5}{2},-1}^{+}\right)}^{2}e^{-\beta \varepsilon_{\frac{5}{2},-1}^{+}}\right), \end{array}$$

(A58) $$\begin{array}{cc} \rho_{6,6}\; & =\;\langle \frac{1}{2},-\frac{5}{2}|\hat{\rho }|\frac{1}{2},-\frac{5}{2}\rangle \;=\;\frac{1}{Z}\left({\left(c_{\frac{5}{2},-2}^{-}\right)}^{2}e^{-\beta \varepsilon_{\frac{5}{2},-2}^{-}}\;+\;{\left(c_{\frac{5}{2},-2}^{+}\right)}^{2}e^{-\beta \varepsilon_{\frac{5}{2},-2}^{+}}\right), \end{array}$$

(A59) $$\begin{array}{cc} \rho_{7,7}\; & =\;\langle -\frac{1}{2},\frac{5}{2}|\hat{\rho }|-\frac{1}{2},\frac{5}{2}\rangle \;=\;\frac{1}{Z}\left({\left(c_{\frac{5}{2},2}^{+}\right)}^{2}e^{-\beta \varepsilon_{\frac{5}{2},2}^{-}}\;+\;{\left(c_{\frac{5}{2},2}^{-}\right)}^{2}e^{-\beta \varepsilon_{\frac{5}{2},2}^{+}}\right), \end{array}$$

(A60) $$\begin{array}{cc} \rho_{8,8}\; & =\;\langle -\frac{1}{2},\frac{3}{2}|\hat{\rho }|-\frac{1}{2},\frac{3}{2}\rangle \;=\;\frac{1}{Z}\left({\left(c_{\frac{5}{2},1}^{+}\right)}^{2}e^{-\beta \varepsilon_{\frac{5}{2},1}^{-}}\;+\;{\left(c_{\frac{5}{2},1}^{-}\right)}^{2}e^{-\beta \varepsilon_{\frac{5}{2},1}^{+}}\right), \end{array}$$

(A61) $$\begin{array}{cc} \rho_{9,9}\; & =\;\langle -\frac{1}{2},\frac{1}{2}|\hat{\rho }|-\frac{1}{2},\frac{1}{2}\rangle \;=\;\frac{1}{Z}\left({\left(c_{\frac{5}{2},0}^{+}\right)}^{2}e^{-\beta \varepsilon_{\frac{5}{2},0}^{-}}\;+\;{\left(c_{\frac{5}{2},0}^{-}\right)}^{2}e^{-\beta \varepsilon_{\frac{5}{2},0}^{+}}\right), \end{array}$$

(A62) $$\begin{array}{cc} \rho_{10,10}\; & =\;\langle -\frac{1}{2},-\frac{1}{2}|\hat{\rho }|-\frac{1}{2},-\frac{1}{2}\rangle \;=\;\frac{1}{Z}\left({\left(c_{\frac{5}{2},-1}^{+}\right)}^{2}e^{-\beta \varepsilon_{\frac{5}{2},-1}^{-}}\;+\;{\left(c_{\frac{5}{2},-1}^{-}\right)}^{2}e^{-\beta \varepsilon_{\frac{5}{2},-1}^{+}}\right), \end{array}$$

(A63) $$\begin{array}{cc} \rho_{11,11}\; & =\;\langle -\frac{1}{2},-\frac{3}{2}|\hat{\rho }|-\frac{1}{2},-\frac{3}{2}\rangle \;=\;\frac{1}{Z}\left({\left(c_{\frac{5}{2},-2}^{+}\right)}^{2}e^{-\beta \varepsilon_{\frac{5}{2},-2}^{-}}\;+\;{\left(c_{\frac{5}{2},-2}^{-}\right)}^{2}e^{-\beta \varepsilon_{\frac{5}{2},-2}^{+}}\right), \end{array}$$

(A64) $$\begin{array}{cc} \rho_{12,12}\; & =\;\langle -\frac{1}{2},-\frac{5}{2}|\hat{\rho }|-\frac{1}{2},-\frac{5}{2}\rangle \;=\;\frac{1}{Z}e^{-\beta \varepsilon_{\frac{5}{2},-3}},\\ \rho_{2,7}\; & =\;\langle \frac{1}{2},\frac{3}{2}|\hat{\rho }|-\frac{1}{2},\frac{5}{2}\rangle \;=\;\frac{c_{\frac{5}{2},2}^{+}c_{\frac{5}{2},2}^{-}}{Z}\left(e^{-\beta \varepsilon_{\frac{5}{2},2}^{+}}\;-\;e^{-\beta \varepsilon_{\frac{5}{2},2}^{-}}\right), \end{array}$$

(A65) $$\begin{array}{cc} \rho_{7,2}\; & =\;\langle -\frac{1}{2},\frac{5}{2}|\hat{\rho }|\frac{1}{2},\frac{3}{2}\rangle \;=\;\frac{c_{\frac{5}{2},2}^{+}c_{\frac{5}{2},2}^{-}}{Z}\left(e^{-\beta \varepsilon_{\frac{5}{2},2}^{+}}\;-\;e^{-\beta \varepsilon_{\frac{5}{2},2}^{-}}\right),\\ \rho_{3,8}\; & =\;\langle \frac{1}{2},\frac{1}{2}|\hat{\rho }|-\frac{1}{2},\frac{3}{2}\rangle \;=\;\frac{c_{\frac{5}{2},1}^{+}c_{\frac{5}{2},1}^{-}}{Z}\left(e^{-\beta \varepsilon_{\frac{5}{2},1}^{+}}\;-\;e^{-\beta \varepsilon_{\frac{5}{2},1}^{-}}\right), \end{array}$$

(A66) $$\begin{array}{cc} \rho_{8,3}\; & =\;\langle -\frac{1}{2},\frac{3}{2}|\hat{\rho }|\frac{1}{2},\frac{1}{2}\rangle \;=\;\frac{c_{\frac{5}{2},1}^{+}c_{\frac{5}{2},1}^{-}}{Z}\left(e^{-\beta \varepsilon_{\frac{5}{2},1}^{+}}\;-\;e^{-\beta \varepsilon_{\frac{5}{2},1}^{-}}\right),\\ \rho_{4,9}\; & =\;\langle \frac{1}{2},-\frac{1}{2}|\hat{\rho }|-\frac{1}{2},\frac{1}{2}\rangle \;=\;\frac{c_{\frac{5}{2},0}^{+}c_{\frac{5}{2},0}^{-}}{Z}\left(e^{-\beta \varepsilon_{\frac{5}{2},0}^{+}}\;-\;e^{-\beta \varepsilon_{\frac{5}{2},0}^{-}}\right), \end{array}$$

(A67) $$\begin{array}{cc} \rho_{9,4}\; & =\;\langle -\frac{1}{2},\frac{1}{2}|\hat{\rho }|\frac{1}{2},-\frac{1}{2}\rangle \;=\;\frac{c_{\frac{5}{2},0}^{+}c_{\frac{5}{2},0}^{-}}{Z}\left(e^{-\beta \varepsilon_{\frac{5}{2},0}^{+}}\;-\;e^{-\beta \varepsilon_{\frac{5}{2},0}^{-}}\right),\\ \rho_{5,10}\; & =\;\langle \frac{1}{2},-\frac{3}{2}|\hat{\rho }|-\frac{1}{2},-\frac{1}{2}\rangle \;=\;\frac{c_{\frac{5}{2},-1}^{+}c_{\frac{5}{2},-1}^{-}}{Z}\left(e^{-\beta \varepsilon_{\frac{5}{2},-1}^{+}}\;-\;e^{-\beta \varepsilon_{\frac{5}{2},-1}^{-}}\right), \end{array}$$

(A68) $$\begin{array}{cc} \rho_{10,5}\; & =\;\langle -\frac{1}{2},-\frac{1}{2}|\hat{\rho }|\frac{1}{2},-\frac{3}{2}\rangle \;=\;\frac{c_{\frac{5}{2},-1}^{+}c_{\frac{5}{2},-1}^{-}}{Z}\left(e^{-\beta \varepsilon_{\frac{5}{2},-1}^{+}}\;-\;e^{-\beta \varepsilon_{\frac{5}{2},-1}^{-}}\right),\\ \rho_{6,11}\; & =\;\langle \frac{1}{2},-\frac{5}{2}|\hat{\rho }|-\frac{1}{2},-\frac{3}{2}\rangle \;=\;\frac{c_{\frac{5}{2},-2}^{+}c_{\frac{5}{2},-2}^{-}}{Z}\left(e^{-\beta \varepsilon_{\frac{5}{2},-2}^{+}}\;-\;e^{-\beta \varepsilon_{\frac{5}{2},-2}^{-}}\right), \end{array}$$

(A69) $$\begin{array}{cc} \rho_{11,6}\; & =\;\langle -\frac{1}{2},-\frac{3}{2}|\hat{\rho }|\frac{1}{2},-\frac{5}{2}\rangle \;=\;\frac{c_{\frac{5}{2},-2}^{+}c_{\frac{5}{2},-2}^{-}}{Z}\left(e^{-\beta \varepsilon_{\frac{5}{2},-2}^{+}}\;-\;e^{-\beta \varepsilon_{\frac{5}{2},-2}^{-}}\right). \end{array}$$

The partition function Z of the mixed spin-(1/2,5/2) Heisenberg dimer reads as follows

(A70) $$\begin{array}{cc} Z & \;=\;2\left\{e^{-\frac{5\beta }{4}\left(J+5D\right)}\cosh\left(3\beta h\right)\;+\;2e^{\frac{\beta }{4}\left(J-17D\right)}\cosh\left(\frac{\beta }{2}\sqrt{4{\left(J\;-\;2D\right)}^{2}\;+\;5{\left(J\Delta \right)}^{2}}\right)\cosh\left(2\beta h\right)\right.\\ \; & \;+\;2e^{\frac{\beta }{4}\left(J-5D\right)}\cosh\left(\frac{\beta }{2}\sqrt{{\left(J\;-\;2D\right)}^{2}\;+\;8{\left(J\Delta \right)}^{2}}\right)\cosh\left(\frac{\beta h}{2}\right)\;+\;\left.e^{\frac{\beta }{4}\left(J-D\right)}\cosh\left(\frac{3\beta }{2}\sqrt{{\left(J\Delta \right)}^{2}}\right)\right\}. \end{array}$$

Finally, the probability coefficients $c_{S,S_{t}^{z}}^{\pm }$ and the respective energies $\varepsilon_{S,S_{t}^{z}}^{\pm }$ ($S_{t}^{z}=-3$, −2, −1, 0, 1, 2, 3) have the explicit forms

(A71) $$\begin{array}{cc} c_{\frac{5}{2},-2}^{\mp } & \;=\;\frac{1}{\sqrt{2}}\sqrt{1\;\pm \;\frac{2(J\;-\;2D)}{\sqrt{4{\left(J\;-\;2D\right)}^{2}\;+\;5{\left(J\Delta \right)}^{2}}}},\;\varepsilon_{\frac{5}{2},-2}^{\mp }\;=\;-\frac{1}{4}\left(J\;-\;17D\;-\;8h\right)\;\mp \;\frac{1}{2}\sqrt{4{\left(J\;-\;2D\right)}^{2}\;+\;5{\left(J\Delta \right)}^{2}}, \end{array}$$

(A72) $$\begin{array}{cc} c_{\frac{5}{2},-1}^{\mp } & \;=\;\frac{1}{\sqrt{2}}\sqrt{1\;\pm \;\frac{\left(J\;-\;2D\right)}{\sqrt{{\left(J\;-\;2D\right)}^{2}\;+\;8{\left(J\Delta \right)}^{2}}}},\;\varepsilon_{\frac{5}{2},-1}^{\mp }\;=\;-\frac{1}{4}\left(J\;-\;5D\;-\;4h\right)\;\mp \;\frac{1}{2}\sqrt{{\left(J\;-\;2D\right)}^{2}\;+\;8{\left(J\Delta \right)}^{2}}, \end{array}$$

(A73) $$\begin{array}{cc} c_{\frac{5}{2},0}^{\mp } & \;=\;\frac{1}{\sqrt{2}},\;\varepsilon_{\frac{5}{2},0}^{\mp }\;=\;-\frac{1}{4}\left(J\;-\;D\right)\;\mp \;\frac{3}{2}\sqrt{{\left(J\Delta \right)}^{2}}, \end{array}$$

(A74) $$\begin{array}{cc} c_{\frac{5}{2},1}^{\mp } & \;=\;\frac{1}{\sqrt{2}}\sqrt{1\;\mp \;\frac{\left(J\;-\;2D\right)}{\sqrt{{\left(J\;-\;2D\right)}^{2}\;+\;8{\left(J\Delta \right)}^{2}}}},\;\varepsilon_{\frac{5}{2},1}^{\mp }\;=\;-\frac{1}{4}\left(J\;-\;5D\;+\;4h\right)\;\mp \;\frac{1}{2}\sqrt{{\left(J\;-\;2D\right)}^{2}\;+\;8{\left(J\Delta \right)}^{2}}, \end{array}$$

(A75) $$\begin{array}{cc} c_{\frac{5}{2},2}^{\mp } & \;=\;\frac{1}{\sqrt{2}}\sqrt{1\;\mp \;\frac{2(J\;-\;2D)}{\sqrt{4{\left(J\;-\;2D\right)}^{2}\;+\;5{\left(J\Delta \right)}^{2}}}},\;\varepsilon_{\frac{5}{2},2}^{\mp }\;=\;-\frac{1}{4}\left(J\;-\;17D\;+\;8h\right)\;\mp \;\frac{1}{2}\sqrt{4{\left(J\;-\;2D\right)}^{2}\;+\;5{\left(J\Delta \right)}^{2}}, \end{array}$$

(A76) $$\begin{array}{cc} c_{\frac{5}{2},\pm 3} & \;=\;1,\;\varepsilon_{\frac{5}{2},\pm 3}\;=\;\frac{5}{4}\left(J\;+\;5D\right)\;\mp \;3h. \end{array}$$

## Appendix B. The Explicit Form of the Eigenvalues $\lambda_{S_{tm}^{z}}$

The explicit form of eigenvalues $\lambda_{S_{tm}^{z}}$ ($S_{tm}^{z}\;=\;-S\;+\;1/2,\ldots ,S\;-\;1/2$) of the partially transposed density matrix $\rho^{T_{1/2}}$ (25) is as follows

if $S_{tm}^{z}\;=\;-S\;+\;1/2$:

(A77)
$$\begin{array}{cc} \lambda_{S_{tm}^{z}}^{\mp } & \;=\;\frac{1}{2Z}\left\{{\left(c_{S,S_{tm}^{z}+1}^{-}\right)}^{2}e^{-\beta \varepsilon_{S,S_{tm}^{z}+1}^{-}}\;+\;{\left(c_{S,S_{tm}^{z}+1}^{+}\right)}^{2}e^{-\beta \varepsilon_{S,S_{tm}^{z}+1}^{+}}\;+\;e^{-\beta \varepsilon_{S,-(S+\frac{1}{2})}}\right\}\\ \; & \;\mp \;\frac{1}{2Z}\left\{{\left[{\left(c_{S,S_{tm}^{z}+1}^{-}\right)}^{2}e^{-\beta \varepsilon_{S,S_{tm}^{z}+1}^{-}}\;+\;{\left(c_{S,S_{tm}^{z}+1}^{+}\right)}^{2}e^{-\beta \varepsilon_{S,S_{tm}^{z}+1}^{+}}\;-\;e^{-\beta \varepsilon_{S,-(S+\frac{1}{2})}}\right]}^{2}\right.\\ \; & +\;{\left.4{\left[c_{S,S_{tm}^{z}}^{-}c_{S,S_{tm}^{z}}^{+}\left(e^{-\beta \varepsilon_{S,S_{tm}^{z}}^{-}}\;-\;e^{-\beta \varepsilon_{S,S_{tm}^{z}}^{+}}\right)\right]}^{2}\right\}}^{1/2}. \end{array}$$

if $S_{tm}^{z}\;=\;S\;-\;1/2$:

(A78)
$$\begin{array}{cc} \lambda_{S_{tm}^{z}}^{\mp } & \;=\;\frac{1}{2Z}\left\{e^{-\beta \varepsilon_{S,(S+\frac{1}{2})}}\;+\;{\left(c_{S,S_{tm}^{z}-1}^{+}\right)}^{2}e^{-\beta \varepsilon_{S,S_{tm}^{z}-1}^{-}}\;+\;{\left(c_{S,S_{tm}^{z}-1}^{-}\right)}^{2}e^{-\beta \varepsilon_{S,S_{tm}^{z}-1}^{+}}\right\}\\ \; & \;\mp \;\frac{1}{2Z}\left\{{\left[e^{-\beta \varepsilon_{S,(S+\frac{1}{2})}}\;-\;{\left(c_{S,S_{tm}^{z}-1}^{+}\right)}^{2}e^{-\beta \varepsilon_{S,S_{tm}^{z}-1}^{-}}\;-\;{\left(c_{S,S_{tm}^{z}-1}^{-}\right)}^{2}e^{-\beta \varepsilon_{S,S_{tm}^{z}-1}^{+}}\right]}^{2}\right.\\ \; & +\;{\left.4{\left[c_{S,S_{tm}^{z}}^{-}c_{S,S_{tm}^{z}}^{+}\left(e^{-\beta \varepsilon_{S,S_{tm}^{z}}^{-}}\;-\;e^{-\beta \varepsilon_{S,S_{tm}^{z}}^{+}}\right)\right]}^{2}\right\}}^{1/2}. \end{array}$$

if $S_{tm}^{z}\;=\;-S\;+\;3/2,\ldots ,S\;-\;3/2$:

(A79)
$$\begin{array}{cc} \lambda_{S_{tm}^{z}}^{\mp } & \;=\;\frac{1}{2Z}\left\{{\left(c_{S,S_{tm}^{z}+1}^{-}\right)}^{2}e^{-\beta \varepsilon_{S,S_{tm}^{z}+1}^{-}}\;+\;{\left(c_{S,S_{tm}^{z}+1}^{+}\right)}^{2}e^{-\beta \varepsilon_{S,S_{tm}^{z}+1}^{+}}\right.\\ \; & \;\;+\;\left.{\left(c_{S,S_{tm}^{z}-1}^{+}\right)}^{2}e^{-\beta \varepsilon_{S,S_{tm}^{z}-1}^{-}}\;+\;{\left(c_{S,S_{tm}^{z}-1}^{-}\right)}^{2}e^{-\beta \varepsilon_{S,S_{tm}^{z}-1}^{+}}\right\}\\ \; & \;\mp \;\frac{1}{2Z}\left\{\left[{\left(c_{S,S_{tm}^{z}+1}^{-}\right)}^{2}e^{-\beta \varepsilon_{S,S_{tm}^{z}+1}^{-}}\;+\;{\left(c_{S,S_{tm}^{z}+1}^{+}\right)}^{2}e^{-\beta \varepsilon_{S,S_{tm}^{z}+1}^{+}}\;-\;{\left(c_{S,S_{tm}^{z}-1}^{+}\right)}^{2}e^{-\beta \varepsilon_{S,S_{tm}^{z}-1}^{-}}\right.\right.\\ \; & \;{\left.\;-{\left(c_{S,S_{tm}^{z}-1}^{-}\right)}^{2}e^{-\beta \varepsilon_{S,S_{tm}^{z}-1}^{+}}\right]}^{2}\;+\;{\left.4{\left[c_{S,S_{tm}^{z}}^{-}c_{S,S_{tm}^{z}}^{+}\left(e^{-\beta \varepsilon_{S,S_{tm}^{z}}^{-}}\;-\;e^{-\beta \varepsilon_{S,S_{tm}^{z}}^{+}}\right)\right]}^{2}\right\}}^{1/2}. \end{array}$$

## Appendix C. Density Matrices Partially Transposed with Respect the Spin-1/2 Subsystem

### Appendix C.1. Partially Transposed Density Matrix of the Mixed Spin-(1/2,1) Heisenberg Dimer

The block diagonal structure of the partially transposed density matrix with respect to the spin-1/2 subsystem of the spin-(1/2,1) Heisenberg dimer reads

(A80) $$\begin{array}{c} {\hat{\rho }}^{T_{1/2}}\;=\;\begin{array}{cc} & \begin{array}{cccccc} |\frac{1}{2},\;-\;1\rangle & |\;-\;\frac{1}{2},1\rangle & |\frac{1}{2},1\rangle & |\;-\;\frac{1}{2},0\rangle & |\frac{1}{2},0\rangle & |\;-\;\frac{1}{2},\;-\;1\rangle \end{array}\\ \begin{array}{c} \langle \frac{1}{2},\;-\;1|\;\;\;\\ \langle \;-\;\frac{1}{2},1|\;\;\;\\ \langle \frac{1}{2},1|\;\;\;\\ \langle \;-\;\frac{1}{2},0|\;\;\;\\ \langle \frac{1}{2},0|\;\;\;\\ \langle \;-\;\frac{1}{2},\;-\;1|\;\;\; \end{array} & \left(\begin{array}{c} \begin{array}{cccccc} \rho_{3,3} & \;0 & \;0 & \;0 & \;0 & \;0\\ 0 & \;\rho_{4,4} & \;0 & \;0 & \;0 & \;0\\ 0 & \;0 & \;\rho_{1,1} & \;\rho_{4,2} & \;0 & \;0\\ 0 & \;0 & \;\rho_{2,4} & \;\rho_{5,5} & \;0 & \;0\\ 0 & \;0 & \;0 & \;0 & \;\rho_{2,2} & \;\rho_{5,3}\\ 0 & \;0 & \;0 & \;0 & \;\rho_{3,5} & \;\rho_{6,6} \end{array} \end{array}\right) \end{array}\;\;. \end{array}$$

The non-zero elements of $\rho_{i,j}^{T_{1/2}}$ are explicitly defined in Equations (A2)–(A9).

### Appendix C.2. Partially Transposed Density Matrix of the Mixed Spin-(1/2,3/2) Heisenberg Dimer

The block diagonal structure of the partially transposed density matrix with respect to the spin-1/2 subsystem of the spin-(1/2,3/2) Heisenberg dimer reads

(A81) $$\begin{array}{c} {\hat{\rho }}^{T_{1/2}}\;=\;\begin{array}{cc} & \begin{array}{cccccccc} |\frac{1}{2},\;-\;\frac{3}{2}\rangle & |\;-\;\frac{1}{2},\frac{3}{2}\rangle & |\frac{1}{2},\frac{3}{2}\rangle & |\;-\;\frac{1}{2},\frac{1}{2}\rangle & |\frac{1}{2},\frac{1}{2}\rangle & |\;-\;\frac{1}{2},\;-\;\frac{1}{2}\rangle & |\frac{1}{2},\;-\;\frac{1}{2}\rangle & |\;-\;\frac{1}{2},\;-\;\frac{3}{2}\rangle \end{array}\\ \begin{array}{c} \langle \frac{1}{2},\;-\;\frac{3}{2}|\;\;\;\\ \langle \;-\;\frac{1}{2},\frac{3}{2}|\;\;\;\\ \langle \frac{1}{2},\frac{3}{2}|\;\;\;\\ \langle \;-\;\frac{1}{2},\frac{1}{2}|\;\;\;\\ \langle \frac{1}{2},\frac{1}{2}|\;\;\;\\ \langle \;-\;\frac{1}{2},\;-\;\frac{1}{2}|\;\;\;\\ \langle \frac{1}{2},\;-\;\frac{1}{2}|\;\;\;\\ \langle \;-\;\frac{1}{2},\;-\;\frac{3}{2}|\;\;\; \end{array} & \left(\begin{array}{cccccccc} \rho_{4,4} & \;0 & \;0 & \;0 & \;0 & \;0 & \;0 & \;0\\ 0 & \;\rho_{5,5} & \;0 & \;0 & \;0 & \;0 & \;0 & \;0\\ 0 & \;0 & \;\rho_{1,1} & \;\rho_{5,2} & \;0 & \;0 & \;0 & \;0\\ 0 & \;0 & \;\rho_{2,5} & \;\rho_{6,6} & \;0 & \;0 & \;0 & \;0\\ 0 & \;0 & \;0 & \;0 & \;\rho_{2,2} & \;\rho_{6,3} & \;0 & \;0\\ 0 & \;0 & \;0 & \;0 & \;\rho_{3,6} & \;\rho_{7,7} & \;0 & \;0\\ 0 & \;0 & \;0 & \;0 & \;0 & \;0 & \;\rho_{3,3} & \;\rho_{7,4}\\ 0 & \;0 & \;0 & \;0 & \;0 & \;0 & \;\rho_{4,7} & \;\rho_{8,8} \end{array}\right) \end{array}\;\;. \end{array}$$

The non-zero elements of $\rho_{i,j}^{T_{1/2}}$ are explicitly defined in Equations (A15)–(A25).

### Appendix C.3. Partially Transposed Density Matrix of the Mixed Spin-(1/2,2) Heisenberg Dimer

The block diagonal structure of the partially transposed density matrix with respect to the spin-1/2 subsystem of the spin-(1/2,2) Heisenberg dimer reads

(A82) $$\begin{array}{c} {\hat{\rho }}^{T_{1/2}}\;=\;\begin{array}{cc} & \begin{array}{cccccccccc} |\frac{1}{2},\;-\;2\rangle & |\;-\;\frac{1}{2},2\rangle & |\frac{1}{2},2\rangle & |\;-\;\frac{1}{2},1\rangle & |\frac{1}{2},1\rangle & |\;-\;\frac{1}{2},0\rangle & |\frac{1}{2},0\rangle & |\;-\;\frac{1}{2},\;-\;1\rangle & |\frac{1}{2},\;-\;1\rangle & |\;-\;\frac{1}{2},\;-\;2\rangle \end{array}\\ \begin{array}{c} \langle \frac{1}{2},\;-\;2|\;\;\;\\ \langle \;-\;\frac{1}{2},2|\;\;\;\\ \langle \frac{1}{2},2|\;\;\;\\ \langle \;-\;\frac{1}{2},1|\;\;\;\\ \langle \frac{1}{2},1|\;\;\;\\ \langle \;-\;\frac{1}{2},0|\;\;\;\\ \langle \frac{1}{2},0|\;\;\;\\ \langle \;-\;\frac{1}{2},\;-\;1|\;\;\;\\ \langle \frac{1}{2},\;-\;1|\;\;\;\\ \langle \;-\;\frac{1}{2},\;-\;2|\;\;\; \end{array} & \left(\begin{array}{cccccccccc} \rho_{5,5} & \;0 & \;0 & \;0 & \;0 & \;0 & \;0 & \;0 & \;0 & \;0\\ 0 & \;\rho_{6,6} & \;0 & \;0 & \;0 & \;0 & \;0 & \;0 & \;0 & \;0\\ 0 & \;0 & \;\rho_{1,1} & \;\rho_{6,2} & \;0 & \;0 & \;0 & \;0 & \;0 & \;0\\ 0 & \;0 & \;\rho_{2,6} & \;\rho_{7,7} & \;0 & \;0 & \;0 & \;0 & \;0 & \;0\\ 0 & \;0 & \;0 & \;0 & \;\rho_{2,2} & \;\rho_{7,3} & \;0 & \;0 & \;0 & \;0\\ 0 & \;0 & \;0 & \;0 & \;\rho_{3,7} & \;\rho_{8,8} & \;0 & \;0 & \;0 & \;0\\ 0 & \;0 & \;0 & \;0 & \;0 & \;0 & \;\rho_{3,3} & \;\rho_{8,4} & \;0 & \;0\\ 0 & \;0 & \;0 & \;0 & \;0 & \;0 & \;\rho_{4,8} & \;\rho_{9,9} & \;0 & \;0\\ 0 & \;0 & \;0 & \;0 & \;0 & \;0 & \;0 & \;0 & \;\rho_{4,4} & \;\rho_{9,5}\\ 0 & \;0 & \;0 & \;0 & \;0 & \;0 & \;0 & \;0 & \;\rho_{5,9} & \;\rho_{10,10} \end{array}\right) \end{array}\;\;. \end{array}$$

The non-zero elements of $\rho_{i,j}^{T_{1/2}}$ are explicitly defined in Equations (A32)–(A45).

### Appendix C.4. Partially Transposed Density Matrix of the Mixed Spin-(1/2,5/2) Heisenberg Dimer

The block diagonal structure of the partially transposed density matrix with respect to the spin-1/2 subsystem of the spin-(1/2,5/2) Heisenberg dimer reads

(A83) $$\begin{array}{c} {\hat{\rho }}^{T_{1/2}}\;=\;\begin{array}{cc} & \begin{array}{cccccccccccc} |\frac{1}{2},\;-\;\frac{5}{2}\rangle & |\;-\;\frac{1}{2},\frac{5}{2}\rangle & |\frac{1}{2},\frac{5}{2}\rangle & |\;-\;\frac{1}{2},\frac{3}{2}\rangle & |\frac{1}{2},\frac{3}{2}\rangle & |\;-\;\frac{1}{2},\frac{1}{2}\rangle & |\frac{1}{2},\frac{1}{2}\rangle & |\;-\;\frac{1}{2},\;-\;\frac{1}{2}\rangle & |\frac{1}{2},\;-\;\frac{1}{2}\rangle & |\;-\;\frac{1}{2},\;-\;\frac{3}{2}\rangle & |\frac{1}{2},\;-\;\frac{3}{2}\rangle & |\;-\;\frac{1}{2},\;-\;\frac{5}{2}\rangle \end{array}\\ \begin{array}{c} \langle \frac{1}{2},\;-\;\frac{5}{2}|\;\;\;\\ \langle \;-\;\frac{1}{2},\frac{5}{2}|\;\;\;\\ \langle \frac{1}{2},\frac{5}{2}|\;\;\;\\ \langle \;-\;\frac{1}{2},\frac{3}{2}|\;\;\;\\ \langle \frac{1}{2},\frac{3}{2}|\;\;\;\\ \langle \;-\;\frac{1}{2},\frac{1}{2}|\;\;\;\\ \langle \frac{1}{2},\frac{1}{2}|\;\;\;\\ \langle \;-\;\frac{1}{2},\;-\;\frac{1}{2}|\;\;\;\\ \langle \frac{1}{2},\;-\;\frac{1}{2}|\;\;\;\\ \langle \;-\;\frac{1}{2},\;-\;\frac{3}{2}|\;\;\;\\ \langle \frac{1}{2},\;-\;\frac{3}{2}|\;\;\;\\ \langle \;-\;\frac{1}{2},\;-\;\frac{5}{2}|\;\;\; \end{array} & \left(\begin{array}{c} \begin{array}{cccccccccccc} \rho_{6,6} & \;0 & \;0 & \;0 & \;0 & \;0 & \;0 & \;0 & \;0 & \;0 & \;0 & \;0\\ 0 & \;\rho_{7,7} & \;0 & \;0 & \;0 & \;0 & \;0 & \;0 & \;0 & \;0 & \;0 & \;0\\ 0 & \;0 & \;\rho_{1,1} & \;\rho_{7,2} & \;0 & \;0 & \;0 & \;0 & \;0 & \;0 & \;0 & \;0\\ 0 & \;0 & \;\rho_{2,7} & \;\rho_{8,8} & \;0 & \;0 & \;0 & \;0 & \;0 & \;0 & \;0 & \;0\\ 0 & \;0 & \;0 & \;0 & \;\rho_{2,2} & \;\rho_{8,3} & \;0 & \;0 & \;0 & \;0 & \;0 & \;0\\ 0 & \;0 & \;0 & \;0 & \;\rho_{3,8} & \;\rho_{9,9} & \;0 & \;0 & \;0 & \;0 & \;0 & \;0\\ 0 & \;0 & \;0 & \;0 & \;0 & \;0 & \;\rho_{3,3} & \;\rho_{9,4} & \;0 & \;0 & \;0 & \;0\\ 0 & \;0 & \;0 & \;0 & \;0 & \;0 & \;\rho_{4,9} & \;\rho_{10,10} & \;0 & \;0 & \;0 & \;0\\ 0 & \;0 & \;0 & \;0 & \;0 & \;0 & \;0 & \;0 & \;\rho_{4,4} & \;\rho_{10,5} & \;0 & \;0\\ 0 & \;0 & \;0 & \;0 & \;0 & \;0 & \;0 & \;0 & \;\rho_{5,10} & \;\rho_{11,11} & \;0 & \;0\\ 0 & \;0 & \;0 & \;0 & \;0 & \;0 & \;0 & \;0 & \;0 & \;0 & \;\rho_{5,5} & \;\rho_{11,6}\\ 0 & \;0 & \;0 & \;0 & \;0 & \;0 & \;0 & \;0 & \;0 & \;0 & \;\rho_{6,11} & \;\rho_{12,12} \end{array} \end{array}\right) \end{array}\;\;. \end{array}$$

The non-zero elements of $\rho_{i,j}^{T_{1/2}}$ are explicitly defined in Equations (A53)–(A69).
